# Modified full-face snorkel masks as reusable personal protective equipment for hospital personnel

**DOI:** 10.1371/journal.pone.0244422

**Published:** 2021-01-13

**Authors:** Laurel Kroo, Anesta Kothari, Melanie Hannebelle, George Herring, Thibaut Pollina, Ray Chang, Dominic Peralta, Samhita P. Banavar, Eliott Flaum, Hazel Soto-Montoya, Hongquan Li, Kyle Combes, Emma Pan, Khang Vu, Kelly Yen, James Dale, Patrick Kolbay, Simon Ellgas, Rebecca Konte, Rozhin Hajian, Grace Zhong, Noah Jacobs, Amit Jain, Filip Kober, Gerry Ayala, Quentin Allinne, Nicholas Cucinelli, Dave Kasper, Luca Borroni, Patrick Gerber, Ross Venook, Peter Baek, Nitin Arora, Philip Wagner, Roberto Miki, Jocelyne Kohn, David Kohn Bitran, John Pearson, Beatriz Arias-Arco, Ricardo Larrainzar-Garijo, Cristián Muñiz Herrera, Manu Prakash

**Affiliations:** 1 Department of Mechanical Engineering, Stanford University, Stanford, CA, United States of America; 2 Department of Bioengineering, Stanford University, Stanford, CA, United States of America; 3 Swiss Federal Institute of Technology (EPFL), School of Engineering, Lausanne, Switzerland; 4 Swiss Federal Institute of Technology (EPFL), School of Life Sciences, Lausanne, Switzerland; 5 Department of Electrical Engineering, Stanford University, Stanford, CA, United States of America; 6 Stellar Design LLC, San Mateo, CA, United States of America; 7 Stanford University, Biophysics Program, Stanford, CA, United States of America; 8 Olin College of Engineering, Needham, MA, United States of America; 9 Deakin College, Australia; 10 Department of Bioengineering, University of Utah, Salt Lake City, UT, United States of America; 11 Department of Anesthesiology, University of Utah, Salt Lake City, UT, United States of America; 12 Waymo, Mountain View, CA, United States of America; 13 Department of Applied Mathematics, Harvard University, Cambridge, MA, United States of America; 14 Mountain View, CA, United States of America; 15 Independent Contributor, Italy; 16 Wildhorn Outfitters, Draper, Salt Lake City, UT, United States of America; 17 Decathlon (Subea), Product Engineer, Hendaye, France; 18 University of Michigan, Entrepreneurial Leadership Faculty, Ann Arbor, MI, United States of America; 19 iSnorkel Inc, Dexter, Salt Lake City, UT, United States of America; 20 Swiss Federal Institute of Technology (EPFL), Safety, Prevention and Health Domain, Lausanne, Switzerland; 21 U.S. Anesthesia Partners Texas, Dallas, TX, United States of America; 22 University of Alabama at Birmingham, Birmingham, AL, United States of America; 23 Hospital for Special Surgery, New York City, NY, United States of America; 24 Miki & Alfonso Hand & Upper Extremity Center, Miami, FL, United States of America; 25 Instituto de Oftalmologia, Ophthalmologist, Santiago, Chile; 26 Hospital Universitario Infanta Leonor, Madrid, Spain; 27 Anesthesiologist, Santiago, Chile; China University of Mining and Technology, CHINA

## Abstract

Here we adapt and evaluate a full-face snorkel mask for use as personal protective equipment (PPE) for health care workers, who lack appropriate alternatives during the COVID-19 crisis in the spring of 2020. The design (referred to as Pneumask) consists of a custom snorkel-specific adapter that couples the snorkel-port of the mask to a rated filter (either a medical-grade ventilator inline filter or an industrial filter). This design has been tested for the sealing capability of the mask, filter performance, CO2 buildup and clinical usability. These tests found the Pneumask capable of forming a seal that exceeds the standards required for half-face respirators or N95 respirators. Filter testing indicates a range of options with varying performance depending on the quality of filter selected, but with typical filter performance exceeding or comparable to the N95 standard. CO2 buildup was found to be roughly equivalent to levels found in half-face elastomeric respirators in literature. Clinical usability tests indicate sufficient visibility and, while speaking is somewhat muffled, this can be addressed via amplification (Bluetooth voice relay to cell phone speakers through an app) in noisy environments. We present guidance on the assembly, usage (donning and doffing) and decontamination protocols. The benefit of the Pneumask as PPE is that it is reusable for longer periods than typical disposable N95 respirators, as the snorkel mask can withstand rigorous decontamination protocols (that are standard to regular elastomeric respirators). With the dire worldwide shortage of PPE for medical personnel, our conclusions on the performance and efficacy of Pneumask as an N95-alternative technology are cautiously optimistic.

## Introduction

During the COVID-19 crisis in the Spring of 2020, there is a global shortage of personal-protective equipment (PPE) for medical personnel **[1]**. Here we present the concept of an adapted full-face snorkel mask as PPE for this user group, and evaluate its performance for use in a medical setting. This solution was designed as a reusable, stop-gap solution for healthcare workers to help address the short-term global N95 respirator shortage.

The design concept is to connect the top snorkel-port of a recreational full-face snorkel mask to a medically or NIOSH-rated filter, where the snorkel is typically attached. This is done through a simple custom adapter piece, which is injection molded out of a robust medical-grade polypropylene material (but can also be 3D-printed from high-resolution, biocompatible resins for small-volume production). A second, optional adapter piece allows for this port to attach to industrial quarter-turn NIOSH P100 filters, for expanded filter supply-chain access in the event that ventilator inline filters are unavailable or unsuitable for use.

**Fig 1:**
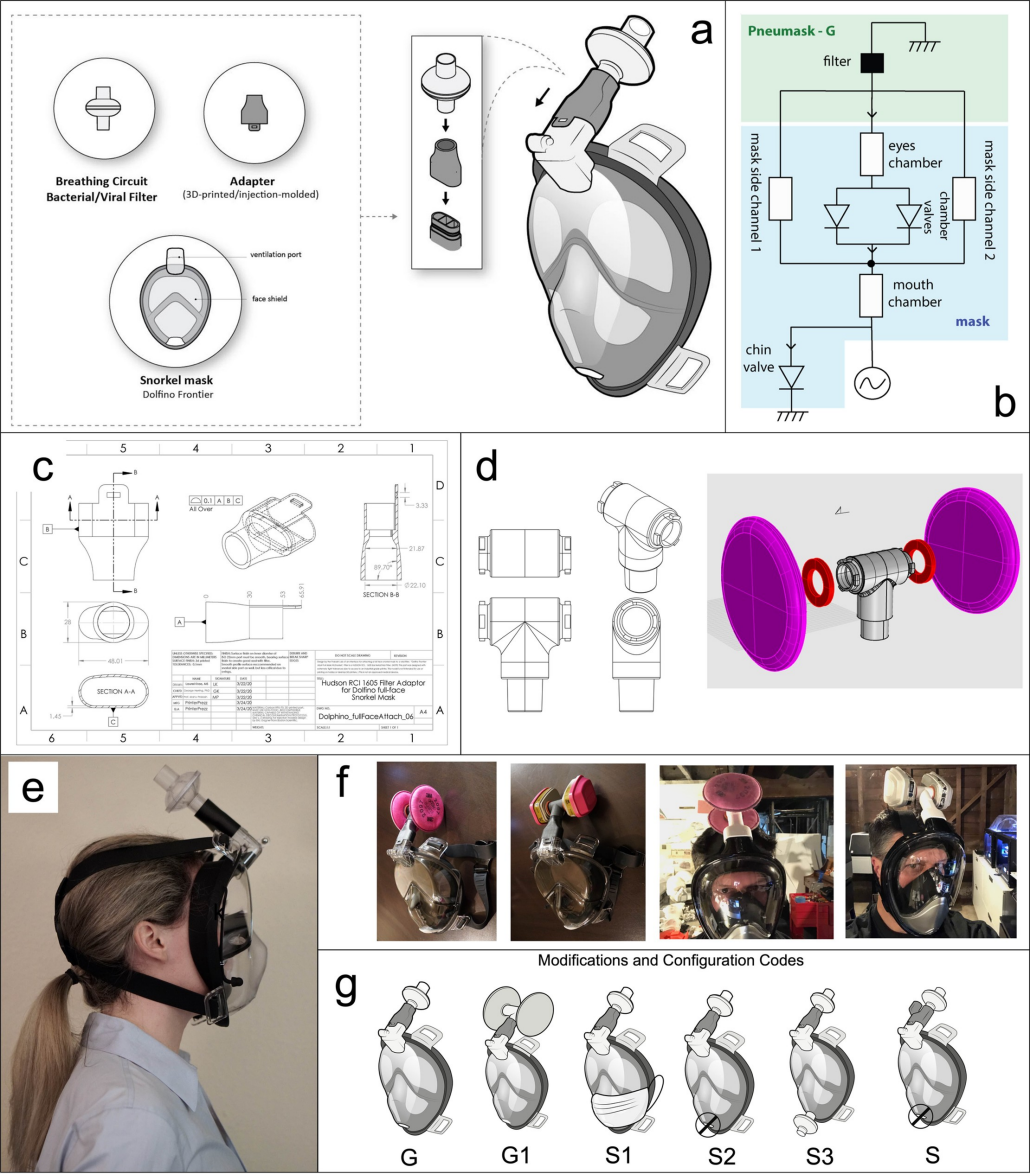
Concept of a modified snorkel-mask or "Pneumask" as PPE. The device consists of a full-face snorkel mask, an adapter, and an FDA or NIOSH-rated filter. b) Air Pathway for the Pneumask. Notation: diodes = valves; ground = atmospheric pressure. c) Original Pneumask adapter from the Prakash Lab was initially designed to be 3D-printed from Carbon RPU-70, a biocompatible resin. Latter versions of the adapter were made and edited by Eric Gagner for injection molding, which allowed for high-tolerance quality parts to be manufactured at high volume. d) A second, option piece designed by Dominic Peralta of Stellar Designs, allows for two quarter-turn NIOSH filters to attach a standard ISO 22mm female port. e) Side view of the Pneumask-G device on an author. f) Industrial filters in the Pneumask-G1 configuration are shown. g) Configurations Pneumask-G and Pneumask-G1 are the main topic of this study. However, we have heard anecdotal reports from the community of people modifying the system to allow for use in "sterile-field" environments, such as by using a surgical mask over the chin valve (S1), taping shut the chin valve (S2), replacement of the chin valve with a second filter (S3), or designing a more complex top adapter part that keeps exhale channels and inhale channels separated; typically also used in conjunction with additional one-way valves. We do not provide recommendations or evaluate the efficacy of Pneumask-S(1–3) designs in this study.

Inhaled and exhaled air are routed through different paths inside an off-the-shelf full-face snorkel mask, as pictured in Fig 1. The inhaled air passes through the center of 3 channels inside of the top snorkel port, then through the eye-chamber, and then through a set of internal one-way valves into the mouth chamber where it is then expelled by the user. There are two possible routes for the expelled air: it can be exhaled directly out of the mask through a one-way valve in the mouth region of the mask (“chin valve”), and it can also be routed through soft tubing side-channels that connect the mouth region directly to the other two channels in the snorkel-port at the top of the mask (which route the exhaled air out through the filter).

The stock configuration of most full-face masks allows the exhalation to follow both of these routes; the volumetric proportion of air routed through the chin-valve versus the exhale channels is a weak function of the filter's pneumatic resistance. Higher resistance filters cause a slightly larger proportion of exhale air to be routed through the chin-valve, lower resistance filters cause slightly more air to be routed through the exhale channels. For the Hudson RCI viral filter, our models indicate approximately 91% of flow is directed through the chin valve and 9% is exhaled up through the filter.

We are aware of many alternative design configurations that have been proposed by the global community. The system is sensitive to modification, and performance evaluations in this paper are specific to the simplest configuration, referred to as “Pneumask-G” and “Pneumask-G1” in Fig 1G. Our team focused on the evaluation of this configuration, as it balances functional performance with mechanical simplicity for mass-scale implementation. Many of the other configurations in Fig 1G are aimed at blocking or filtering air from “chin-valve” to allow for use in surgical environments where a “sterile-field” is necessary. These are a minority of use-case scenarios, and intubation procedures done by anesthesiologists on COVID-19 patients do not typically require a sterile field **[2].** Redirecting exhaled air through the filter rather than the chin-valve can slightly increase the CO2 concentration and substantially increase fogging issues in the eye chamber due to a build-up of exhaled vapor. Thus, while we briefly discuss other configurations, they are not evaluated extensively, and we focus on the 'Pneumask-G' configuration herein.

The subject of this study is specific to the use of these masks as PPE for doctors, nurses and hospital-affiliated staff. These hospital personnel have substantially higher risk of infection than civilians, due to their repeated exposure in a high-risk environment **[3,4]**. Additionally, the medical respiratory filter supply has long been restricted to direct-purchasing by hospital-affiliates. At the time of this publication, the industrial filter supply in the USA has also been redirected primarily to this purchasing group (such as the P100 3M elastomeric respirator filters used for the construction industry). Uncoordinated consumer demand on medical respiratory filters could cause further shortages of these filters in hospitals (that are also needed for intubated patients and general anesthesia). Snorkeling masks have also been used in the context of the COVID-19 pandemic for patient therapy, in conjunction with Bilevel Positive Airway Pressure (BiPAP) systems **[5]**. However, this report is specific to the use of these masks as passive personal protective equipment (for hospital staff), without any additional pressure sources other than the user’s lungs (such as a positive pressure PPE or patient therapy applications).

We recognize that under normal circumstances, the adaptation of recreational sports equipment for medical usage would not be advisable, because of the availability of superior alternatives (more medically-specific in their overall design, such as NIOSH-approved full-face respirators or PAPRs). However, a recent survey **[6]** shows that in the US, 31.4% of health care workers reported that there are no masks in their hospitals. The number of healthcare workers without access to suitable PPE will continue to grow if no additional efforts are made, due to a discrepancy between demand and available supply. With lack of PPE solutions for health care workers globally, novel and creative alternatives must be explored to bridge this short-term gap in supply. But even more critical than the design of these new solutions, is the timely, rigorous, and stringent evaluation of their quantitative performance. The FDA in the United States has cleared NIOSH industrial half-face elastomeric respirators (with exhale valves) for use in medical environments [7,8]. This study aims to quantitatively compare the performance of modified snorkel-mask respiratory PPE to the standards of disposable N95 respirators (FDA) and half-face elastomeric respirators (NIOSH). Here we share results on topics ranging from CO2 accumulation, fit testing, filtration efficiency testing, exhalation valve performance and clinical usability of this system.

## Methods and results

There are several topics to address on the question of performance and overall efficacy of this design. Please reference the methods section and the supplementary documents for further information on each of these topics.

### Fit testing

We have performed extensive fit testing to evaluate the capability of these masks to perform a seal to the user’s face. Redundant tests were performed at several separate institutions (Stanford Prakash Lab, Stanford Environmental Health and Safety Department, EPFL in Switzerland, and University of Utah), and several were conducted by independent third-parties. Fit testing was performed with a total of 6 participants (3 individuals for qualitative testing and 3 different individuals for quantitative testing). The purpose of this fit testing study is not to prove that this mask will fit everyone in the general population (which would require hundreds of participants to statistically prove)—but rather to evaluate if these masks are generally are capable of forming the quality of seal necessary for a PPE that is comparable to half-face elastomeric respirators (no gross leakage due to facemask construction methods).

**Fig 2:**
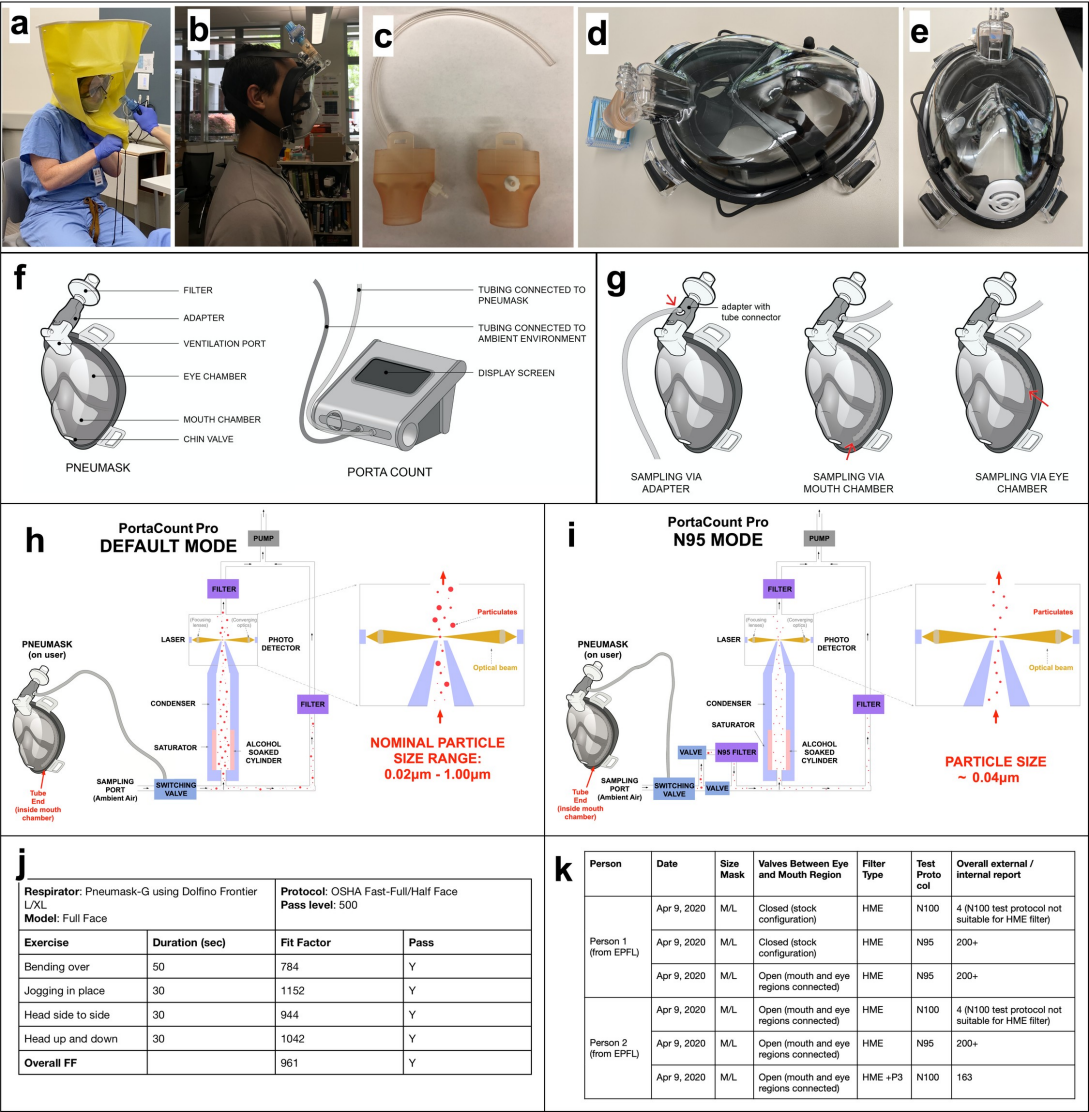
Fit testing. a) Qualitative fit testing being performed in the Department of Anesthesiology at the University of Utah. b) Qualitative fit testing performed in Prakash Lab at Stanford University. c) modified adapter to use in conjunction with PortaCount devices for quantitative fit testing d-e) Mask with modified adapter and with internal measurement tube routed down through exhale channel. f) Pneumask and PortaCount device basic terminology g) Sampling tube positions can be at the adapter, in the mouth chamber or in the eye chamber. Most conservative tests are done with the sampling tube in the mouth chamber h)Typical operating mode of the PortaCount machine quantitative fit tests. May be used if a HEPA-rated filter is available. i) N95 mode is to be used if a viral filter is used that may have <99% efficiency at peak breathing flow rates. Note that some ventilator inline filters fall into this category. j) Testing completed at Stanford by the occupational health and safety department. k) Testing completed at EPFL by the authors to compare performance to half-face respirators.

We conducted both qualitative and quantitative fit tests. Both types of tests suggest that Pneumasks create an excellent seal to the face, equivalent to half-face elastomeric respirators, and nominally superior to disposable N95s (Fig 2).

Qualitative fit testing involves a liquid aerosol with a sweaty or bitter taste generated within a confinement around the head of the mask wearer (Fig 2A) **[9]**. At the University of Utah, we performed a qualitative fit test on 3 separate volunteers, utilizing a 3D printed version of our adapter and both an HME anesthesia circuit filter and a HEPA anesthesia circuit filter on a Dolfino Frontier mask. Out of 3 volunteers, 2 were male and 1 was female, and both males had failed their fit test in the past using regular N95 respirators. The fit test was performed by the standard University of Utah Operating Room team as for N95 tests, as part of the emergency COVID-19 response in order to evaluate emergency countermeasure personal protective equipment. Importantly, all 3 individuals passed the fit tests. This practical result indicates that the fit seal satisfies the minimum requirements for an N95 respirator or elastomeric respirator.

For quantitative fit testing, the method used is based on particle counting outside and inside the mask in parallel using the TSI PortaCount device (Fig 2F and 2I) [9]. The ratio of particulates measured outside the mask versus inside gives the fit factor. Certain scores are given for a range of activities, including normal breathing, deep breathing, nodding and shaking of the head, talking, bending down and moderate exercise. Threshold scores are required by different regulatory bodies to “pass” these tests.

Our tests found the Pneumask capable of forming a seal that exceeds the standards required for half-face respirators or N95 respirators, by both US and European standards (Fig 2J and 2K). We found this to be the case for both AquaLeisure’s Dolfino Frontier and the Subea Decathlon (V1) mask models, for different activities and for different positions of the sampling tube within the mask (in the adapter, in the eye chamber, in the mouth chamber). We encourage readers who wish to replicate these fit-testing protocols to reference our methods sections in the supplementary documents on fit testing, and perform quantitative fit testing with a very high performance HEPA-rated inline filter (standard is 99.97% efficient for particles at 0.3 μm). This is to ensure that the fit of the mask (*sealing capability of the mask*) is evaluated separately from the filtration performance. If no filter is available that operates >99% efficiency at breathing flow-rates, you may need to use specialized modes for fit testing (such as N95 mode on PortaCount devices).

### Filter selection and filtration efficiency

Filter testing indicates a range of options with varying performance depending on the quality of filter selected, but with typical filter performance exceeding or comparable to the N95 standard. If multiple filter options are available to clinicians, we recommend the usage of inline pleated, hydrophobic mechanical filters (such as the Pall BB25 or BB50T) rather than electrostatic/viral filters **[10]**. This is due to superior stated filtration performance in the specification of the filter, and also due to superior filter durability. Extended use of filters in this context is under evaluation, with most filters having between a 12–24 hour lifespan, and some having up to a 7-day continuous use lifespan. In S1 File, see Tables S1 and S8 in S1 File in for a summary of ventilator inline filters, listed by manufacturer's specification on filtration efficiency at 0.3 μm particle diameter.

**Fig 3:**
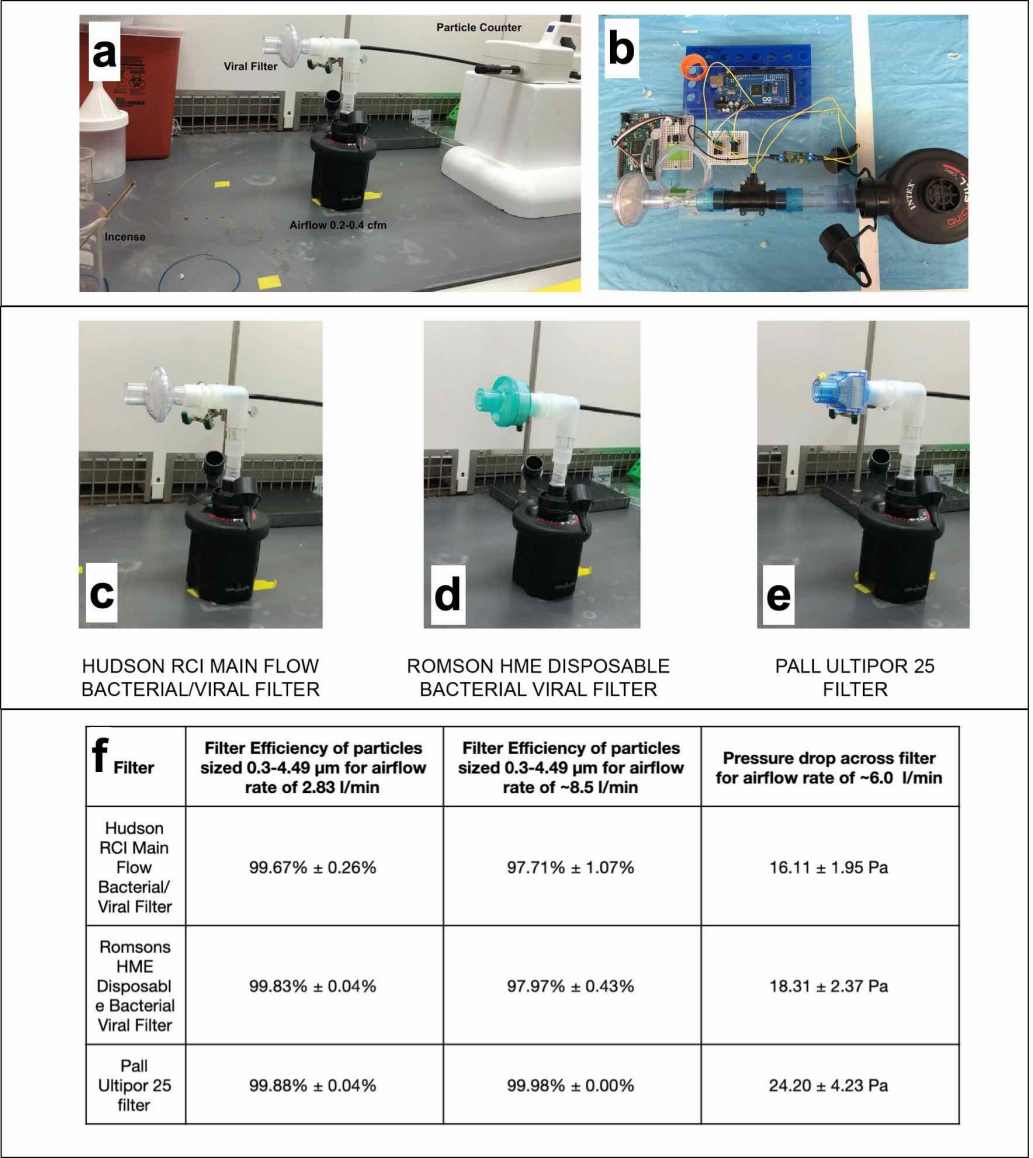
Filter efficiency and pressure drop testing. a) Setup for measuring filter efficiency. b) Setup for measuring pressure drop across filter. c-e) Variety of filters tested, using the setup in a) and b). f) Filter efficiency and pressure drop across filters.

The Stanford lab has also built a low-cost setup for the evaluation and testing of filtration efficiency of filters at different flow rates, as depicted in Fig 3.

### Work of breath

Work of breath on the overall system was evaluated qualitatively to be comfortable to the user with several different filter types for extended periods (1–3 hours). While not intended for extreme extended usage, durations on the order of 4–6 hours of continuous use in clinical environments have been anecdotally reported by international partners (in Chile), with no reported evidence of hypercapnia, despite a large variety of filters used in these improvised systems.

The pressure drops across most of these FDA-approved respiratory inline filters are relativity low (please reference the supplementary section on filter selection and evaluation), and the P100 industrial filters are already rated as compliant with the even stricter NIOSH-42C FR84 standards (inhalation less than or equal to 343 Pa at 85 liters per min) **[11].** Independent measurements done by the authors in the Stanford Prakash Lab were completed on 3 different medical inline filters at a relatively low flow rate of approximately 6 liters per minute, consistently indicating pressure drops between 16 and 24 pascals for different filter brands and models. Regulatory requirements for specific pressure drop limits at set flow rates vary based on the governing body and the application (industrial versus medical**) [11].**

### CO2 species transport and accumulation

**Experimental investigations.** The accumulation of carbon dioxide in the deadspace is a valid concern that can result in significant risks to healthcare workers utilizing respirators **[12]**. The accumulation of CO2 can vary greatly in snorkel masks as the connections between the inhalation and exhalation arms, the design of one-way valves, and possibly attached cartridges are very versatile. Also, the issue of CO2 buildup is not unique to full face mask snorkels nor to elastomeric respirators, but is a known factor in the continuous use of disposable N95 respirators as well [9]. For example, Lim et al., 2006 found that up to 13 of healthcare workers in the SARS outbreak reported headaches during use of N95 (presumably from hypercapnia) and that 4 hours of continuous use of N95 is associated with headaches [12].

**Fig 4:**
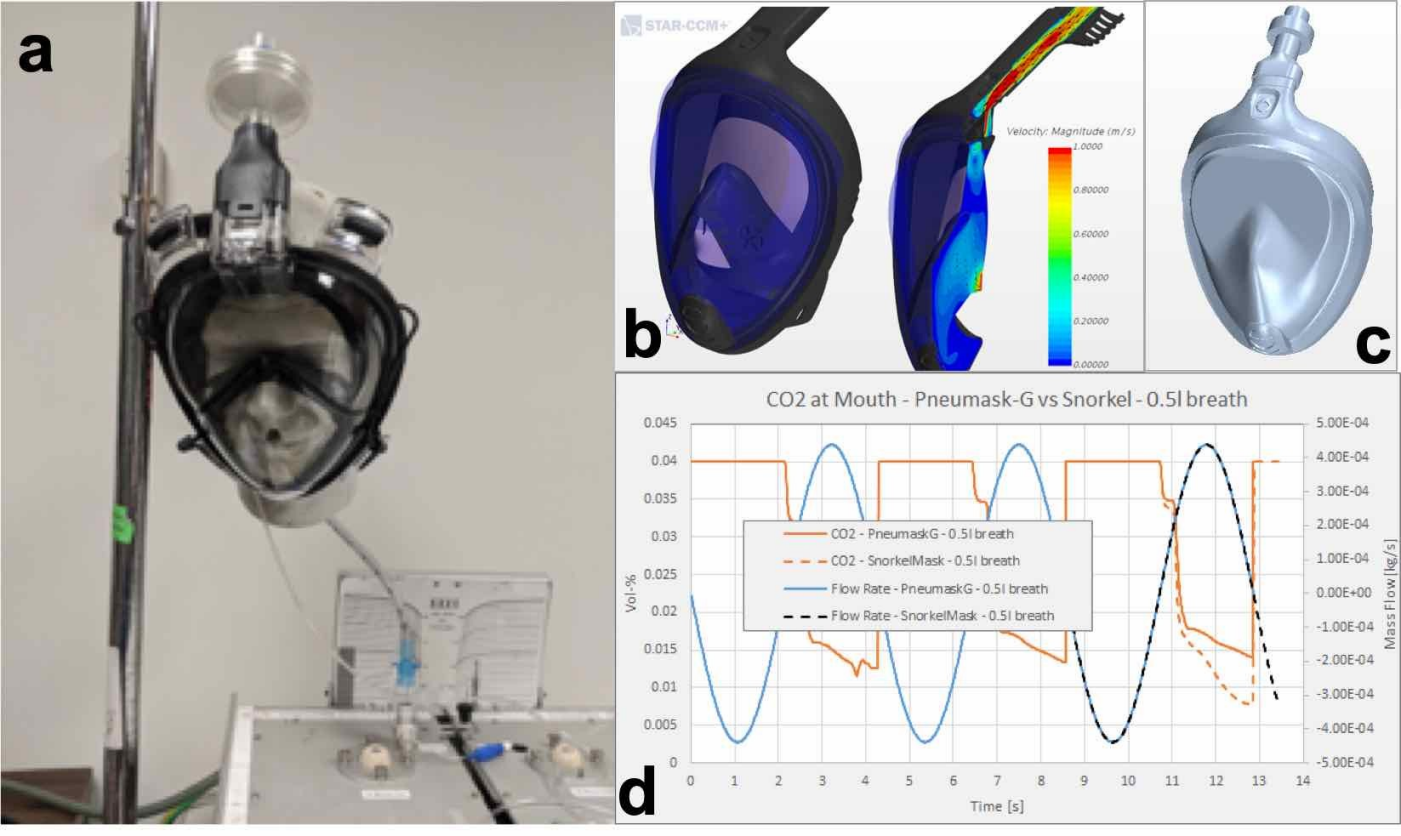
CO2 accumulation testing and CFD simulation. a) Carbon dioxide washout testing utilizing a headform and simulated lung. b) Visualization of flow velocities within the Decathlon Easy Breath mask in stock configuration. c) Pneumask system with adapter and inline filter added. d) Preliminary CFD results on CO2 species transport, comparing the Pneumask with filter resistance to the stock configuration of the mask.

We have completed CO2 testing at University of Utah on a headform and simulated lung apparatus (Fig 4A), and found CO2 levels to be between 1 and 2 percent, which is comparable to levels found in half-face elastomeric respirators in literature [13]. Additionally, one of the authors from University of Utah self-tested and reported that the work of breathing appears similar to an N95 respirator when either filter is attached. Subjectively, it appears comfortable but took a small adjustment period to adapt to breathing to a comfortable level. At this time, we would recommend a periodic (every 5–10 minutes) deep forced exhalation to purge the mask of any CO2 buildup, which is similar to what was previously proposed for elastomeric respirators [9].

To test for data on CO2 accumulation, we utilized the mask-adapter-filter setup attached to a headform and simulated lung (Fig 4A) [14,15]. Carbon dioxide is added to the test lung at rates ranging from 200–500 mL/minute to simulate a range of metabolic output. The test lung was ventilated at respiratory rates of 12–28 breaths/minute, and tidal volumes of 400–600 mL. Gas sampling is performed with a Datex-Ohmeda gas bench at the mouth of the headform after the carbon dioxide concentration in the snorkel mask reaches steady state for at least 4 minutes. The 3 anesthesia circuit filters we tested are Teleflex Main Flow Bacterial/Viral Filter 1605, ISO-GARD Filters and Filter HMEs 28012, and Romsons HME Disposable Bacterial/Viral Filter GS-2095.

In the CO2 accumulation result, for the 3 anesthesia circuit filters we tested, the steady state CO2 concentration inside the mask is approximately 1–2%, which is generally safe for short term usage [14] and comparable to commercial elastomeric respirators [13]. These preliminary results are comparable to our user feedback from University of Utah, where one of our authors self-tested and reported that the work of breathing appears similar to an N95 respirator when either filter is attached. Subjectively, it appeared comfortable but took a small adjustment period to adapt to breathing to a comfortable level. At this time, we would recommend a periodic (every 5–10 minutes) deep forced exhalation to purge the mask of any CO2 buildup, which is quite similar to previously proposed solution for elastomeric respirators [9].

As the risk of CO2 accumulation is directly related to the volume of dead-space, we performed direct volume measurement on our Pneumask-G setting with a Body Glove snorkel mask. The effective dead-space volume of the adapter is 10 mL while the effective dead-space volume of a Virex N100 In-line Filter is 11 mL, adding to a total volume of 21 mL, which is very low compared to the effective dead-space volume of the supplied snorkel (157mL, isolating inhalation/exhalation pathways). These results suggested that if a snorkel manufacturer has passed a CO2 accumulation test with their mask and snorkel tube, it is very likely that a Pneumask-G based on their snorkel mask will also pass a CO2 accumulation test.

Under the same logic, it was proposed that during relaxed or resting states, insufficient quenching of the mask secondary to the low minute ventilation expected may be conducive to CO2 accumulation. The data is available in the supplementary document on CO2 data. We note an increase in the inspired CO2, consistent with rebreathing and insufficient quenching of exhaled CO2, with a maximum value of 9 mmHg of inspired CO2 at the minute 15 of the test, but the EtCO2 values remained stable, as did all the other vital signs values for the entirety of the test. Our explanation is that the inspired CO2 rise triggered an increase in minute ventilation and respiratory rate, maintaining EtCO2 within the normal range. In conclusion, after 80 minutes of mask use under resting conditions, there was no significant accumulation of CO2 and no deleterious effects secondary to the observed elevation of inspired CO2. This suggests that any CO2 accumulation under resting conditions will likely be minimal and automatically adjusted by the user through normal physiologic response to CO2 buildup. A similar user test was performed under strenuous physical activity. The results indicate that in exertion simulating that of most healthcare work, the change in inspiratory CO2 throughout use of the device is negligible and in line with NIOSH standards [9]. Subjective comfort/discomfort was rated from 1 (complete discomfort) to 10 (complete comfort). It is notable that this never fell below a rating of a 7. Further, the volunteer had an appropriate heart rate response for the level of exertion and no further alterations in physiological processes were noted. This indicates the device performs similar to elastomeric respirators under near identical conditions.

#### Transport species modelling

To further support these experiments, we also completed a full CO2 species transport simulation using computation fluid dynamics (Fig 4B–4D). This was also in an effort to better understand CO2 buildup with different flow path configurations in different masks. This model is approximate, and should be used to assess theoretical relative performance of different configurations; not to stipulate absolute or predictive values of species transport without reference to a baseline.

The software used was Siemen’s STAR-CCM+; the model used the segregated flow implicit unsteady solver, with the realizable k-Epsilon URANS turbulence model. Computational runtime is around 8–9 hours per exhale-inhale cycle. We are currently solving these tests using 32 cores Intel XEON on a desktop machine (not on a compute server). Mesh size is 4.8M cells, so we have 150k cells per core. The timestep is set at 0.01s, to compromise between compute speed and quality, with a CFL number around 20. These methods are industry-standard.

One-way valve modeling poses some challenges numerically in the model: to avoid the numerical cost of mesh motion, and the very thin gaps present during opening and closing of the purge valves, the valves are modeled by simply varying the porous resistance of a porous region at the location of the valve. Therefore, the viscous resistance is set to a very high value to force the flow to practically zero when air would flow against the valve's direction. For flow in the valve's direction, the resistance is set to a value that reproduces the pressure drop across the valve in its full-open position. To be clear, the variable porous resistance is currently not set based on the local flow field at each valve, which would be more physical, but caused substantial numerical instability. Instead, each valve's resistance parameter is set based on the global direction of the flow (inhaling, vs exhaling). This approach is only valid since the flow changes direction almost instantaneously throughout the computational domain. While this solution is pragmatic, and allows us to perform the desired qualitative ranking of the CO2 buildup of different mask configurations with quick turn-around, it can indeed be further improved.

These computational predictions of CO2, flow rates and pressures, while they appear similar in range to experiments we report here, we caution readers that these computational models were built specifically to assess relative performance of different design configurations (different flow paths, mask models, etc.) and not absolute values that could be compared to experimental tests.

We are currently extending these preliminary results to ask specific, targeted questions about relative performance of different configurations. Initial results comparing CO2 percentage between a stock-snorkel mask and the Pneumask (with filter and adapter connected) are shown in Fig 4D.

Our simulation results indicated that the average CO2 concentration was found to be 1.87%. Blocking the chin-valve and without any further air path modifications (directing all exhalation through the filter at the top of the mask) increases this to level to approximately 1.96%, according to simulations. These CO2 concentrations are well below the concentration of 5% that would be concerning with regards to hypercapnia [16,17], but it does exceed the 1% CO2 clearance requirement of several international regulatory bodies such as FFP2 (Europe EN 149–2001), KN95 (China GB2626-2006), and P2 (Australia/New Zealand AS/NZA 1716:2012)). Notably, the United States NIOSH-42CFR84 requirements do not stipulate this CO2 requirement, and regulatory-cleared elastomeric respirators currently used in the United States do currently operate in this 1–2 percent CO2 range [18].

Additionally, the model was used to evaluate the effect of filter variety with differences in back-pressure on the airflow within the mask. In this way, we modelled if the choice of filter may influence the airflow pathway substantially enough to influence CO2 concentration or fit. However, the preliminary findings from our model show that the fraction of air that is exhaled through the filter (approximately 9% of total exhale) varies by just 2–3 percent from the baseline, (with a range of filter backpressures tested, that approximately model the pressure-flow dynamics of the three filters we have tested and discussed in the filter testing section). These findings suggest that the configuration is robust to filter choice, with different filter resistances only affecting the airflow dynamics to a minimal extent. See the S1 **File**, Section 1.1 for further detail on the topic.

### Exhalation valve performance

For use in a medical environment, the exhalation valve (“chin valve”) on this system was extensively tested on different mask models in a variety of conditions, and has consistently been found to satisfy NIOSH performance standards for leak rate under suction pressure **[19]**. This is notable, since these masks are fundamentally recreational sports equipment, designed for use underwater. As these snorkel valves are not medically approved, it is necessary to compare the performance of these snorkel valves to NIOSH-rated valves on industrial N95 respirators (the latter of which have been allowable for use in non-sterile field medical environments during this crisis).

The NIOSH standard “Exhalation Valve Leakage Test” (Section 84.182) stipulates the pressures and flow rates necessary for equivalent performance standard to typical N95 respirators. Particularly, it states that: “(a) Dry exhalation valves and valve seats will be subjected to a suction of 25mm water-column height while in normal operating position. (b) Leakage between the valve and valve seat shall not exceed 30 milliliters per minute” [19].

With this specification in mind, we conducted a series of tests to determine technical equivalency to NIOSH standards.

**Fig 5:**
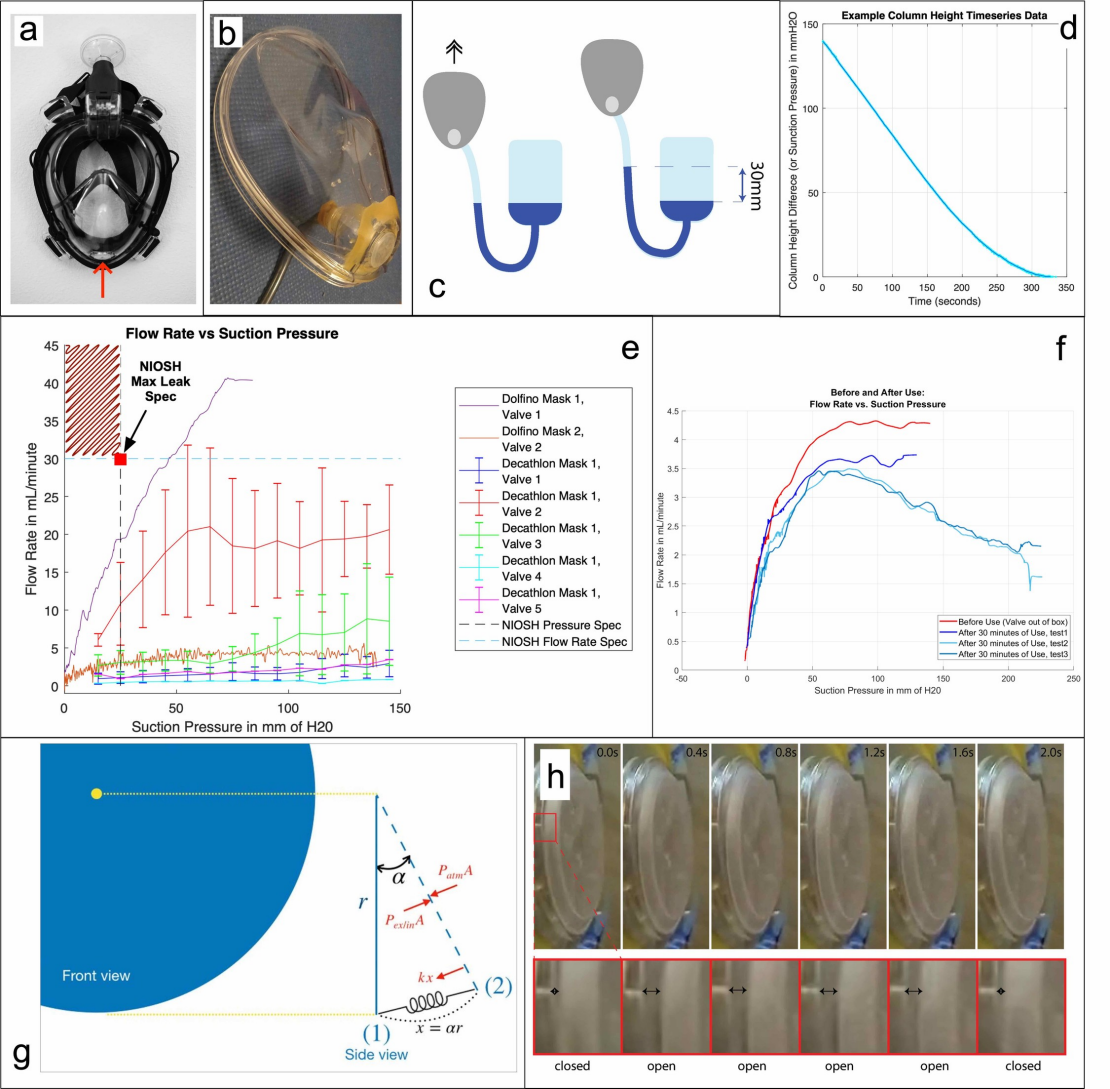
Exhalation valve performance testing. a) Exhalation valve on mask. b) Setup for measuring the leak flow of the chin valve of a Decathlon FreeBreath mask, showing the connection to an 8mm tube. c) Connection to an open water tank—system at equilibrium (chin valve opened). The mask is lifted up (chin valve closed) to create a water column of 30mm, which means that a negative pressure of 30mmH2O is applied to the chin valve. d) an example time series curve of water column height in experiments described in c). e) exhalation valve leakage test results of 2 valves from Dolfino Frontier and 5 valves from Decathlon, compared with the NIOSH standard as designated in red square. All valves passed the NIOSH standard. f) Flow rate versus suction pressure before use (dry valve, as per NIOSH standards) and after use. This test was specifically to test if valve performance suffers in the humid environments of user breathing. To the contrary, evidence suggests that moisture tends to improve valve performance, as shown in this data. g) Schematic of a circular chin valve. The force balance determines the time scale of chin valve closure. h) Time-lapse images of the chin valve opening and closing.

A chamber was glued around the chin valve using epoxy, and a ½” tube (8mm tube for the tests done at EPFL) was connected to this chamber (Fig 5A). The tube was connected to an opened water tank (Fig 5B). Further sealant (either additional epoxy, and also Vaseline) was applied on the connection if necessary to ensure an airtight seal between the measurement tube and the mask. The valve was kept clean of debris, clear of all adhesives and sealants, and dry for testing.

The leak rate of the chin valve at different suction pressures can be computed from a time series of the column height between a reservoir and a water column in the tube. As time passes, the small leakage of the chin valve reduces the negative pressure and therefore the height of the water column. The leak rate can be computed from the geometry of the tube and the derivative of the column height time-series. This holds true because the entire system is quasi-static; there are very minimal inertial effects and the leak rate is slow.

Our results are shown in Fig 5E. We conclude that the leak flow of the exhale valve (chin valve) is consistently lower than the maximum flow allowed to comply with the NIOSH regulation (30mL/min for 25mmH2O of pressure). It is noted that this test was done on a two specific mask models, the Decathlon EasyBreath V1 and the Dolfino Frontier. Although our findings here are reassuring, there is a possibility that different mask models and manufacturers have varying quality of valves, and that quality control within batches may not be as rigorous as it is for airway valves on typical medical devices. For this reason, we recommend testing each mask prior to usage (and especially prior to large-scale usage of any particular mask brand). Gross leaks in valve performance may be identified prior to usage by applying a negative pressure (user inhaling while covering the filter port) and holding for 10 seconds. Unacceptable valve leakage of a defective unit (say, due to a manufacturing error or damaged valve seat) would result in a hissing sound at the chin or a substantial decrease in pressure during the hold period. In addition, qualitative fit testing (as is typically available domestically at hospitals to our users) is also recommended prior to use.

Additionally, we have evaluated the theoretical matter of “valve regurgitation”. This is specifically the behavior of the valve at the transition point between inhale and exhale. We report a theoretical estimation of valve closure time of approximately 0.024 seconds. It is noted that fit testing should indicate if transient-valve regurgitation poses a leakage threat, since these tests were run in use-case contexts with functional valves; fit tests are reassuring on the matter. See supplementary section on further details on transient chin valve modelling.

### Fluid resistance testing

The fluid resistance to projectile synthetic blood of Dolfino Frontier mask and Decathlon EasyBreath v2 mask was tested following the ASTM F1862 protocol as suggested by CDC **[20]**. After calibration of projectile speed, 2 mL of synthetic blood was projected at two speeds (530cm/sec and 635cm/sec) to the surface lens and the chin valve of the 2 snorkel masks. The inner surface was observed for any penetration of synthetic blood.

Both masks passed at both speeds when the synthetic blood was targeting either the lens portion or the chin valve of the snorkel mask. When the chin valve is targeted, although synthetic blood can visually contaminate the chin valve in the Dolfino Frontier mask, no blood was detectable from the inner surface. For Decathlon EasyBreath mask, the chin valve remained clean even if the blood was targeting it. These results showed that Pneumask is safe to use in scenarios where projectile blood is expected. However, if the user is wearing a Pneumask based on a Dolfino Frontier mask, it is still recommended to wash and replace the mask immediately if the chin valve portion is heavily contaminated.

### Clinical usability

Approximately 70 units were distributed to collaborating medical personnel domestically (co-authors), for use in non-sterile, non-infectious environments. Instructions for use were provided with kits of snorkel masks (unopened in-box, unused), couplers and in-line filters. We provided multiple units per medical personnel in different sizes. Decontamination protocols (as given in S1 File, section 1.4) were also provided.

This internal feedback indicated adequate usability, work of breath and sufficient visibility. Some users reported fogging in the eye area of the mask if certain types of filters were used (see Filter Selection section in supplementary). A persistent critique was that speaking can be muffled, especially in noisy environments. In these environments, this muffled speech poses a technical challenge for users, and can interfere with occupational tasks related to communication.

**Fig 6:**
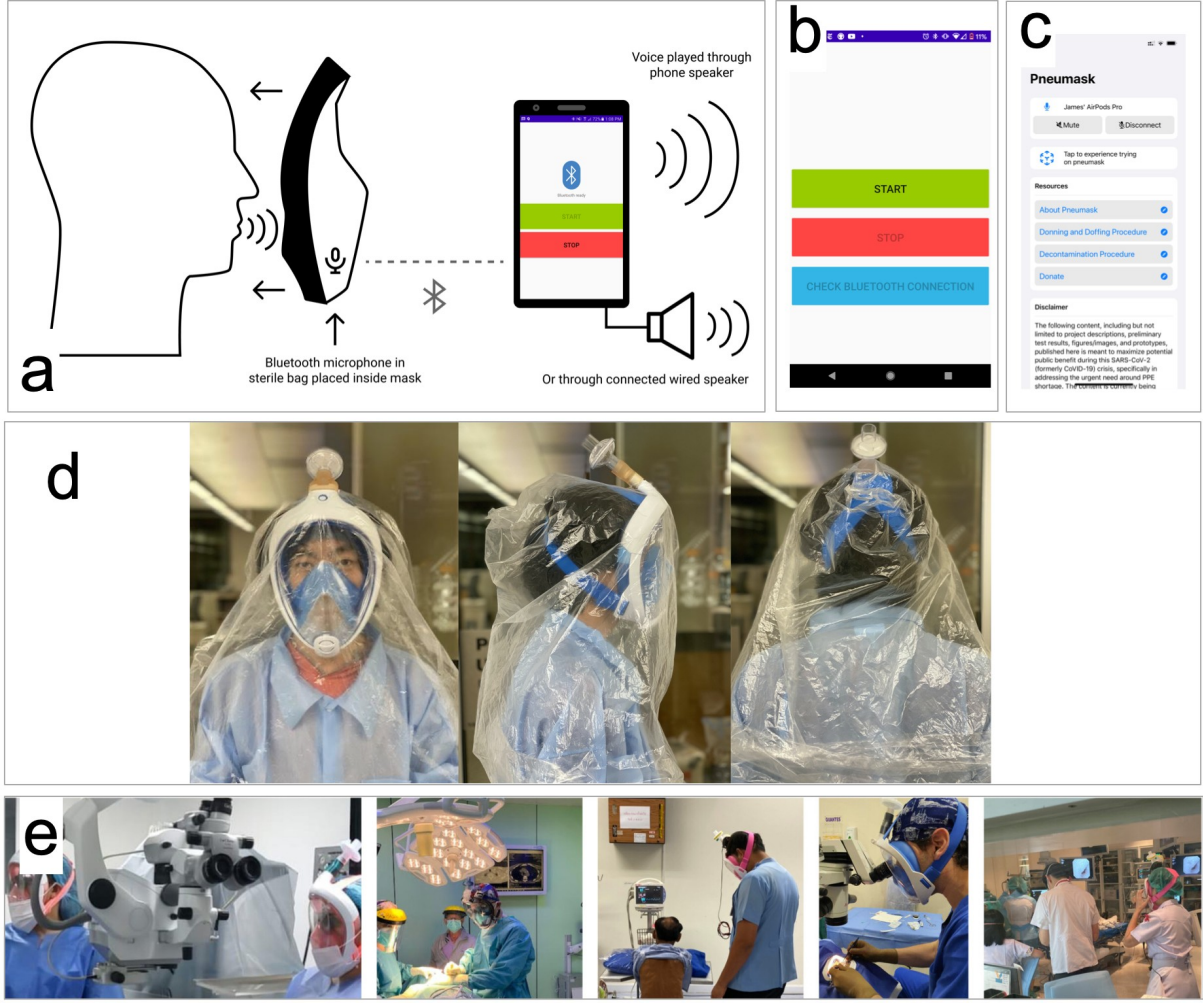
Clinical usability of Pneumask. a)Diagram of Bluetooth amplification solution using only an android cell phone and wireless Bluetooth earbuds placed in the mouth part of the mask (can be placed in a plastic bag to make cleaning easier). b-c) the GUI of our Android app. d) Pneumasks can be used with a disposable plastic hood to offer additional droplet protection. Also shown as references are uses of surgical hood and PAPR [21]. e) Pneumask has been actively used in various healthcare settings.

We have developed an open-source amplification solution (Fig 6A) using hardware that many clinicians own personally: a Bluetooth headset (or earbud) and a smartphone. This solution is optional for users, and will depend on individual preferences and occupational circumstances. The android app is available at the time of publication; the iOS version is still under active development.

### Donning and doffing protocol testing and development

We present guidance on the assembly, filter selection, donning and doffing protocols in our S1 File, Section 1.2 and demonstrated in the attached S1 and S2 Videos. Testing of decontamination protocols indicate reuse of the snorkel masks is technically feasible. Our decontamination guides (S1 File, Section 1.4) should be referenced in detail in the supplementary section. We recommend the mask and adapter be submerged in a bleach solution for decontamination. Other decontamination methods also are feasible on the Dolfino mask—including disinfection with an autoclave.

The filters have limited lifetimes and the number of hours of usage should be tracked and logged, such that use does not exceed the manufacturer's specified lifetime.

Additionally, we recognize that beyond being an alternative to N95 respirators (and protective goggles/face shields), Pneumasks can be combined with a disposable hood to leave no directly exposed hairs or skins that are otherwise susceptible to being contaminated with droplets. Without Pneumasks, this kind of protection is only achievable with PAPR or the use of surgical hood/coveralls, which may also be in short supply and are associated with a more complex doffing process **[21].**

## Discussion and conclusions

### Guidance on use and implementation at scale

**Fig 7:**
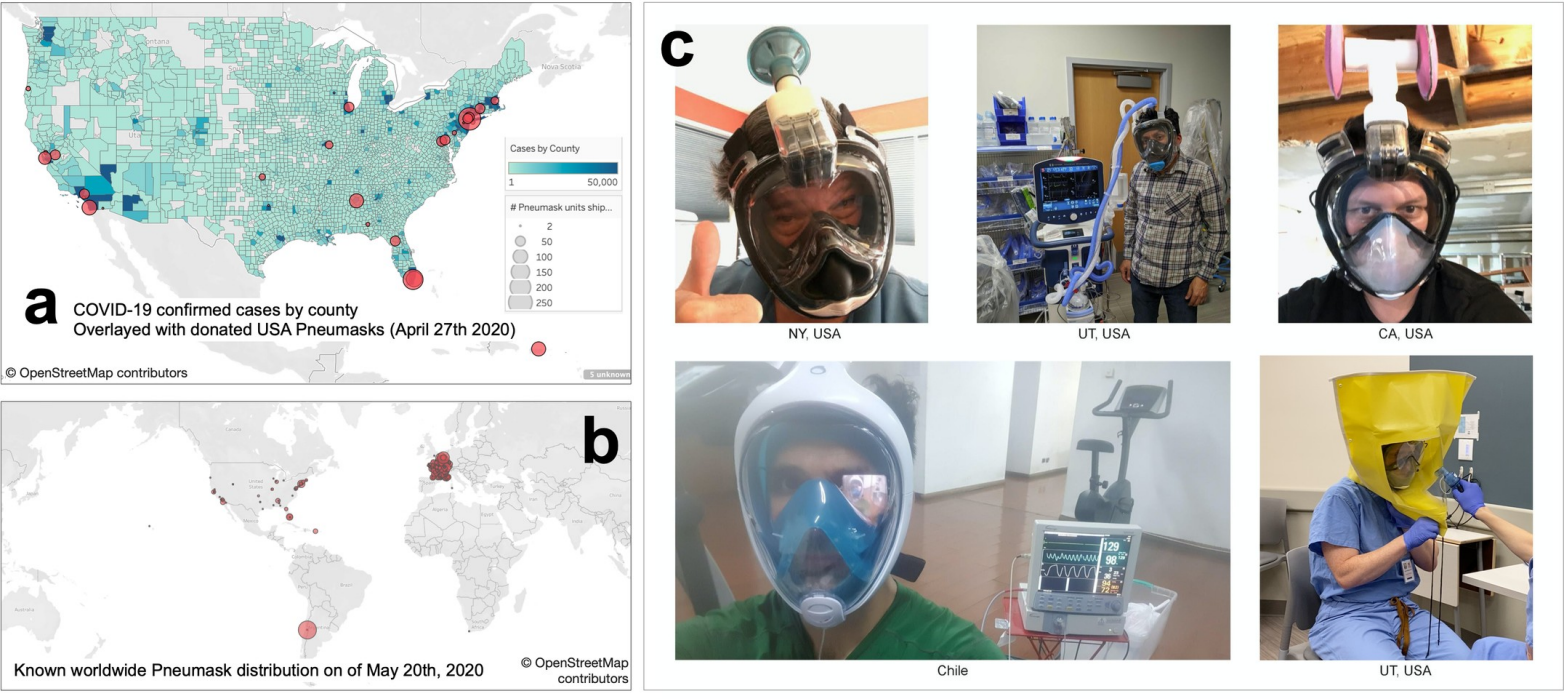
Global efforts that make Pneumask a rigorously tested solution to PPE shortage. a)The donation map of Pneumask(in red circle) overlapped with the case number in the US from April of 2020. Map data in Fig 7A and 7B are from the OpenStreetMap foundation (ODbL 1.0 4.3). Fig 7A and 7B are generated and reprinted from Tableau.com software platform under a CC BY license, with permission from Tableau Software LLC, original copyright 2020. b)The donation map of Pneumask globally. It is approved in France, Belgium and Italy, and the approval is under review in UK, Chile and the USA. c) Collaborators wearing Pneumask.

Our team has been in direct contact with the FDA in the USA, whom we are still in communications with on the topic of formal clearance of this device. This is not yet an FDA-approved medical device for replacement of N95 respirators. The FDA has advised that in the interim, individual clinicians may improvise their own PPE when no suitable FDA-approved alternative is available. We have been advised that the assembly of the mask, adapter and filter by a clinician in the United States constitutes an "improvised PPE device**" [22].** We have been informed that the unmodified snorkeling mask may technically be shipped by distributors to clinicians as a 'face-shield', even prior to official approval. In this manner, clinical users across the USA have been assembling and using these devices beginning in March of 2020 in emergency situations where no suitable alternative was present. As seen in Fig 7A, the COVID-19 case load (by county) correlated well with demand for this device; the red circles are where these devices were deployed on a donation-basis.

Internationally, the Pneumask concept has received formal approval based in part on the data from this international research team (particularly, France received formal approval in early May 2020, sponsored by Decathlon and BIC), followed by Belgium. Discussions are currently ongoing in the UK, Chile and several other countries on the topic of regulatory approval. We are aware of many cooperative efforts to manufacture and distribute these devices at scale in many different countries.

### Outlook

In the context of a dire worldwide shortage of PPE for medical personnel, and where no other approved alternatives are available, we are cautiously optimistic about the performance and efficacy of this system as an effective N95-alternative technology. We have discussed our findings on fit testing, CO2 accumulation, exhalation valve performance, filtration efficiency, work of breath, and clinical usability. Additionally, we have provided guidance on decontamination procedures, and reviewed the continuing joint efforts for implementation at scale.

## Supporting information

S1 File Supplemental materials document. This file contains extensive further methods/process information on testing (Section 1.1), details formal donning and doffing protocols (Section 1.2), provides instruction on mobile app usage for amplification (Section 1.3), details suggested decontamination protocols (Section 1.4), provides a FMEA analysis (Section 1.7), and further provides reference tables and figures in Sections 2 and 3 related to implementation and further study.
(PDF)

S1 Video Author Roberto Miki demonstrates donning protocols. (MP4)

S2 Video Author Roberto Miki demonstrates doffing protocols. (MP4)
