# Supplementary material for: Modified full-face snorkel masks as reusable personal protective equipment for hospital personnel
Source: PLoS One. 2021 Jan 13;16(1):e0244422. doi: 10.1371/journal.pone.0244422 (PMC7806161; doi:10.1371/journal.pone.0244422)
Supplement: S1 File — This file contains extensive further methods/process information on testing (Section 1.1), details formal donning and doffing protocols (Section 1.2), provides instruction on mobile app usage for amplification (Section 1.3), details suggested decontamination protocols (Section 1.4), provides a FMEA analysis (Section 1.7), and further provides reference tables and figures in Sections 2 and 3 related to implementation and further study. (PDF) [file pone.0244422.s001.pdf]

SUPPLEMENTAL MATERIALS: Modified Full-Face Snorkel Masks as Reusable Personal Protective Equipment for Hospital Personnel

Laurel Kroo[1], Anesta Kothari[2], Melanie Hannebelle[3,4], George Herring[2,5], Thibaut Pollina[2], Ray Chang MD[2], Dominic Peralta[6], Samhita P. Banavar[2], Eliott Flaum[7], Hazel Soto-Montoya[2], Hongquan Li[5], Kyle Combes[8], Emma Pan[8], Khang Vu[8], Kelly Yen[8], James Dale , Patrick Kolbay[9,10], Simon Ellgas[11], Rebecca Konte[2], Rozhin Hajian[12], Grace Zhong[2], Noah Jacobs[13], Amit Jain[13], Filip Kober , Gerry Ayala[14], Quentin Allinne[15], Nicholas Cucinelli[16], Dave Kasper[17], Luca Borroni , Patrick Gerber[18], Ross Venook[2], Peter Baek MD[19], Nitin Arora M.D.[20], Philip Wagner MD[21], Roberto Miki MD[22], Jocelyne Kohn MD[23], David Kohn Bitran MD[23], John Pearson MD[12], Beatriz Arias-Arco MD[25], Ricardo Larrainzar-Garijo MD[25], Cristián Muñiz Herrera MD[24], and Manu Prakash[2,*]

1- 25: Affiliations for all authors are listed in the main publication text.

* Corresponding author is Manu Prakash: manup@stanford.edu.

Contents

1 Supplementary Text **2**

1.1 Testing and Ongoing clinical validation . . . 2
Details about Quantitative Fit Test • Details about Filtration Efficiency Testing • Theoretical Estimation of Chin Valve Closure Time • Exercise Test

1.2 Donning and Doffing Procedures . . . 12
Donning Procedures • Doffing Procedures

1.3 Communication App User Instructions . . . 13
Current Android app download instructions • App usage instructions • Troubleshooting

1.4 Decontamination Protocol Testing . . . 14
Autoclaving • Bleach Immersion • Ethanol Immersion • Stretch Test • Dry Heat at 65C

1.5 Decontamination Summary - Guidelines . . . 15

1.6 Suggested Decontamination Protocols . . . 15

1.7 Failure Modes and Effects Analysis (FMEA) . . . 16

2 Supplementary Tables **17**

3 Supplementary Figures **24**

1 Supplementary Text

1.1 Testing and Ongoing clinical validation

1.1.1 Details about Quantitative Fit Test

The primary function of respiratory PPE is to protect the wearer from exposure to pollutants present in air, specifically in this case from particles exhaled/sneezed/coughed by an infected individual. The residual exposure of the wearer depends on three independent additive components: the leak at the face, the penetration through the filter and the internal contamination.

$$\textbf{Residual Exposure} = \textbf{leak at the face} + \textbf{penetration through the filter} + \textbf{internal contamination} \tag{1}$$

The leak at the face depends on how well the mask forms a seal with the wearer face, or said differently, how well the mask fits the wearer face. The fit is clearly dependent on the mask shape and the morphology of the individual face, and should be determined systematically for each wearer with each of the mask models used. As the origin of the leaks is a breakthrough in the sealing, the fit is considered independent from the nature of the pollutant.

The penetration through the filter depends on the efficiency of the filtering material to remove particles from the air. The filtration efficiency is clearly a characteristic of the filter material and the nature of the particle, and is independent from the individual and from the mask shape. The filtration efficiency has to be measured under normalized conditions, or at least with well characterized particles corresponding to the pollutant the wearer has to be protected from. Filters with less than 99% filtration efficiency at inhalation flow rates will interfere with fit tests and can result in a false fit test failure. This problem can be addressed, in quantitative fit testing, by testing masks with lower filtration efficiencies in the N95 mode available in some TSI PortaCount machines.

The internal contamination is mainly due to an inadequate maintenance of the mask, but could be significantly reduced by wearer training, adapted maintenance, and storage protocols (such as sterilization).

Currently, the particles exhaled by the mask wearer have the same size range as those generated by the patients, and which the mask should protect against. The global protection factor of the mask cannot be determined while the mask is being worn, as the wearer-exhaled particles could be misinterpreted as a "leak".

Thus, for multiple scientific reasons, the fit and the filtration efficiency must be determined separately. The exposure of the wearer will be considered adequate when both fit and filtration efficiency criteria are respected.

For their intended purpose, the seal tests of snorkel masks are done by the manufacturing companies underwater. However, the sealing ability of the snorkel masks on dry skin is unknown. Per CDC and NIOSH regulations on the use of elastomeric respirators, a fit test can be performed in the same manner as N95 respirators to ensure seal and safety to use for an individual [19]. At this time, we recommend that all practitioners seeking to utilize these masks perform a fit test under standard N95 fit test conditions.

In addition to this recommendation, additional fit test experiments have been performed in our laboratories. Practically, two types of fit test can be conducted:

- Qualitative fit test: a liquid aerosol with a sweaty or bitter taste are generated within a confinement around the head of the mask wearer. The result of the fit test is based on the detection of the taste under the mask.
- Quantitative fit test: the method is based on a particle counting outside and inside the mask in parallel using the TSI PortaCount device. The ratio out/in gives the fit factor.

As explained above, the fit test is only meant to measure the ability of the mask to form a seal with the wearer's face, and not the efficiency of the respiratory protection. The principle of this test consists of successive measurements of the particulates concentrations inside and outside of the mask during normalized exercises. The ratio between the external and the internal concentrations is called fit factor (FF). The relevancy of the results is dependent on a few assumption:

- The efficiency of the filter is high enough to assure that the particle penetration rate is insignificant compared to the expected leak rate, within the range of the measured particle size ($0.015 - 1$ μm). P3, N100 or HEPA filters are generally required to reach this specification (filtration rate > 99.95 % at 0.3 μm), with a theoretical FF > 2000 in case of perfect fit. For filters with significant penetration rate (P2 or N95), the measure is based on a smaller subset of particle sizes (around 0.04 μm) using the N95 protocol to avoid the counting of the filter-penetrating particles.
- The range of the particle size measures by the PortaCount ($0.015 - 1$ μm) has been selected to stay mostly outside of the range of particulates generated by human exhalation, apart from smokers (it is inadvisable for smokers to perform these tests, but at a minimum, they should not have smoked within 30 minutes of testing). This is necessary because the particulates generated by mask wearer would otherwise be misinterpreted as a leak into the mask.

- The ambient particle count exterior to the mask, in the particle size range measured by the machine, must be significantly higher than the quantity of particles that could be generated by the wearer by any method. Quantitative fit testing units such as the TSI PortaCount are programmed to abort testing if the ambient particle count decreases under a minimum level.

Practically, the quantitative fit test will not measure only the leaks at the wearer's face, but also any leaks in relation with the connection after the filter or with the exhaust valve. In this way, high fit factors reflect both that the leakage at the wearer's face is acceptably low (wearer dependent), but also that the residual leaks at the level of the adapter and the chin valve are acceptable as well (wearer independent).

Here we present quantitative fit test results. We will first discuss the multiple tests run at Stanford University (through multiple groups) on the Dolfino Frontier Mask. We will then review the results both from Stanford and from EPFL on the Subea Decathlon mask. Both mask models passed quantitative fit testing with fit factors that meet the industry-standard threshold for typical half-face elastomeric respirators.

Stanford Prakash Lab Testing: Experiment Parameters

- Fit measured using a PortaCount Pro+
- OSHA Standard
- Half Mask protocol (non-N95)
- 4 candles to provide a constant ambient particle source
- Test Subject: 28 year old male, no facial hair, no history of smoking
- HME HEPA filter (Pall Ultipor 25)
- Snorkel Mask (Dolfino or Decathlon)
- Modified adapter from Formlabs high temperature resin

In order to collect a thorough measurement, each test was repeated in three locations, sampling from the mouth chamber, the eye chamber, and directly from the adapter, right after the filter. This required building two modified adapters. The adapter which sampled directly after the filter was modified by drilling a hole in the adapter between the filter connection port and the mask port. The drilled hole was cleaned and smoothed before a luer lock connector was press fit into the hole and sealed using epoxy. The epoxy was allowed to cure for 24 hours and the assembly was subsequently washed with isopropyl alcohol for 2 minutes. The adapter which sampled from the eye chamber and mouth chamber used a flexible tube to sample air from a desired location. This adapter was modified by drilling a hole large enough for the flexible tube to pass through. The hole was once again cleaned and a luer lock connector was pressed into the tube. The luer lock connector and tube were press fit into the drilled hole and sealed using epoxy. The epoxy was allowed to set for 24 hours before the entire assembly was washed for 2 minutes in isopropyl alcohol. Both adapters were allowed to dry completely before any tests were conducted.

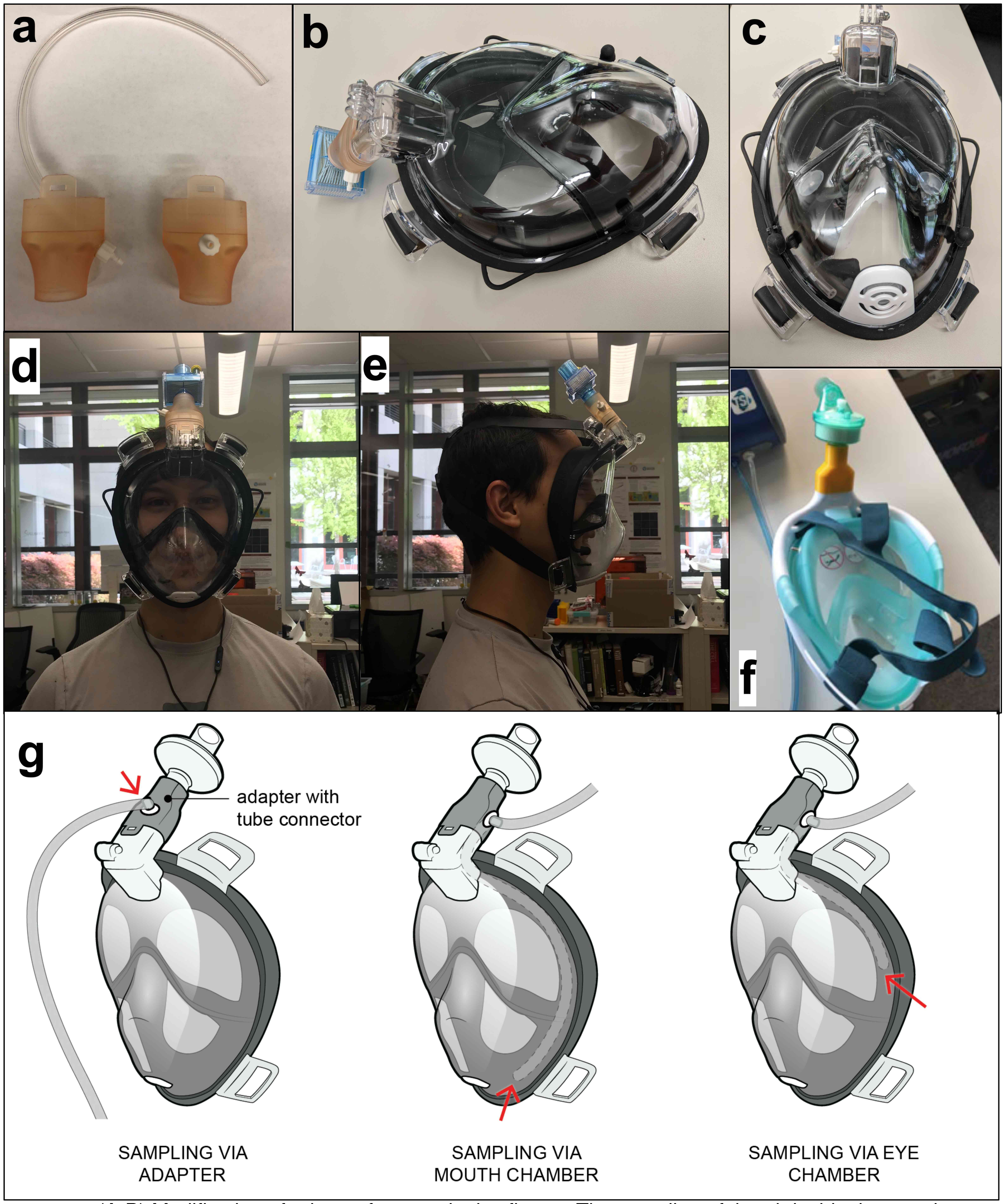

Figure 1 (A-B) Modification of adapter for quantitative fit test. The sampling of the air inside the mouth chamber is achieved by a flexible tubing running through the side channel and connected to the sampling port on the adapter. This method avoids possible deconstructions on the mask surface. (C) The yellow arrow points out the tip ending of the sampling tube. (D) The front view and (E) side view of our testing subject wearing the mask. (F) Subea Decathlon mask used for quantitative fit testing at EPFL. (g) Different testing methods, depicting measuring the exact position for PortaCount sampling tube. Red arrows point to the tube inlet position inside the mask.

Dolfino Frontier Mask Experimental Results Results from the Stanford Prakash Lab are posted here for the fit results on the Dolfino Frontier mask.

	Mouth Chamber	Eye Chamber	Inlet Port
Normal Breathing	482	291	2713
Deep Breathing	133	194	1209
Head Side to Side	439	255	2766
Head Up and Down	257	230	1471
Talking	360	173	698
Bending Over	1104	509	3020
Normal Breathing	477	568	2766
Overall Fit Factor	325	266	1605

Figure 2 Fit factor results when the PortaCount sample tube was connected to the mouth chamber, eye chamber, and inlet port of a Pneumask. This mask consisted of a Dolfino mask connected, via an adapter, to a HEPA rated mechanical HME filter (Pall Ultipor 25). These tests were completed in half-face respirator mode and the mask passed in all three cases.

Further tests were completed independently at Stanford Occupational Health and Safety which confirmed that the Dolfino mask passes the quantitative fit test.

**Respirator**: Pneumask-G using Dolfino Frontier L/XL<br>**Model**: Full Face		**Protocol**: OSHA Fast-Full/Half Face<br>**Pass level**: 500	
**Exercise**	**Duration (sec)**	**Fit Factor**	**Pass**
Bending over	50	784	Y
Jogging in place	30	1152	Y
Head side to side	30	944	Y
Head up and down	30	1042	Y
**Overall FF**		961	Y

Figure 3 Results of quantitative fit tests using PortaCount 8048, conducted by the EHS team at Stanford Health Care. The sample is obtained from the adapter site, using a modified adapter as shown in Figure 1f.

Two additional fit tests were conducted at Stanford Environmental, Health and Safety – one completed using the requirement of a half-face elastomeric respirator and another using the fit factor for a full-face tight fitting Air Purifying Respirator, and the results are shown in Figure 4. The participant (female) wearing Dolfino Frontier with a custom adapter, and a Pall Ultipor 25 breathing filter, who is typically a size M, was still able to pass the quantitative fit test well beyond the requirements on both tests. The minimum passing fit factor was 100 for half-face respirator and 500 for a full-face respirator. The activities that were tested while wearing the mask included bending over (50 seconds), jogging in place (30 seconds), moving head side to side (30 seconds), and moving head up and down (30 seconds), and the fit factor outcomes were all above 750. Please note that, although the test yielded positive results, this was conducted with a limited testing sample and does not yet indicate any certification/endorsement from EH&S of the product. Each entity should also conduct its own evaluation and testing before use.

Subea Decathlon Experimental Results Testing was undertaken both at Stanford University and at EPFL with quantitative fit testing for the Subea Decathlon Mask. Below are the results from our tests at Stanford.

	Mouth Chamber	Inlet Port
Normal Breathing	134	139
Deep Breathing	120	117
Head Side to Side	127	98
Head Up and Down	104	78
Talking	111	116
Bending Over	94	170
Normal Breathing	90	142
Overall Fit Factor	110	116

Figure 4 Fit factor results when the PortaCount sample tube was connected to the mouth chamber and inlet port of a Pneumask. This mask consisted of a Decathlon mask connected, via an adapter, to a HEPA rated mechanical HME filter (Pall Ultipor 25). These tests were completed in half-face respirator mode and the mask passed in all cases.

Important Note: The adapters used in the above test did not fit the Subea mask as well as the Dolfino mask. Tape had to be used to form a better seal. Thus these results should be interpreted as a lower bound on the sealing capabilities of the mask.

Validation of the fit of the Decathlon mask was completed in a separate series of experiments at EPFL, also using a PortaCount Pro+ in N95 mode and following the OSHA 29CFR1910.134 protocol. The Decathlon EasyBreath mask was connected to a medical grade HME filter (DAR adult-pediatric electrostatic filter HME, small) with a 3D-printed PLA connector. The mask was in Pneumask-G configuration (3 snorkel ports connected to the filter, chin valve non modified). The mask was connected through the silicon skirt of the eyes chamber, as indicated in Figure 1f, using the standard connector sold by the manufacturer of the particle counter. In N95 mode, the test was positive for the two individuals (men, freshly shaved) tested, with a fit factor of 200+, which is higher than the requirement for half-masks (100). Removing the chamber valves to connect permanently the eye chamber and the mouth chamber led to the same results. This test was run in N95 mode because the test was completed using an HME filter which was not HEPA rated (fit factors around 4 would have been obtained with the N100 normal protocol under the same testing conditions). The full results from EPFL testing are shown in Figure 5.

Person	Date	Size Mask	Valves Between Eye and Mouth Region	Filter Type	Test Proto col	Overall external / internal report
Person 1 (from EPFL)	Apr 9, 2020	M/L	Closed (stock configuration)	HME	N100	4 (N100 test protocol not suitable for HME filter)
	Apr 9, 2020	M/L	Closed (stock configuration)	HME	N95	200+
	Apr 9, 2020	M/L	Open (mouth and eye regions connected)	HME	N95	200+
Person 2 (from EPFL)	Apr 9, 2020	M/L	Open (mouth and eye regions connected)	HME	N100	4 (N100 test protocol not suitable for HME filter)
	Apr 9, 2020	M/L	Open (mouth and eye regions connected)	HME	N95	200+
	Apr 9, 2020	M/L	Open (mouth and eye regions connected)	HME +P3	N100	163

Figure 5 Fit factor test results from EPFL (translated from French). Note that we have identified a common testing issue mistake – you cannot use the default N100 mode unless the respirator or HME filter is rated above 99% at respiratory flow rates.

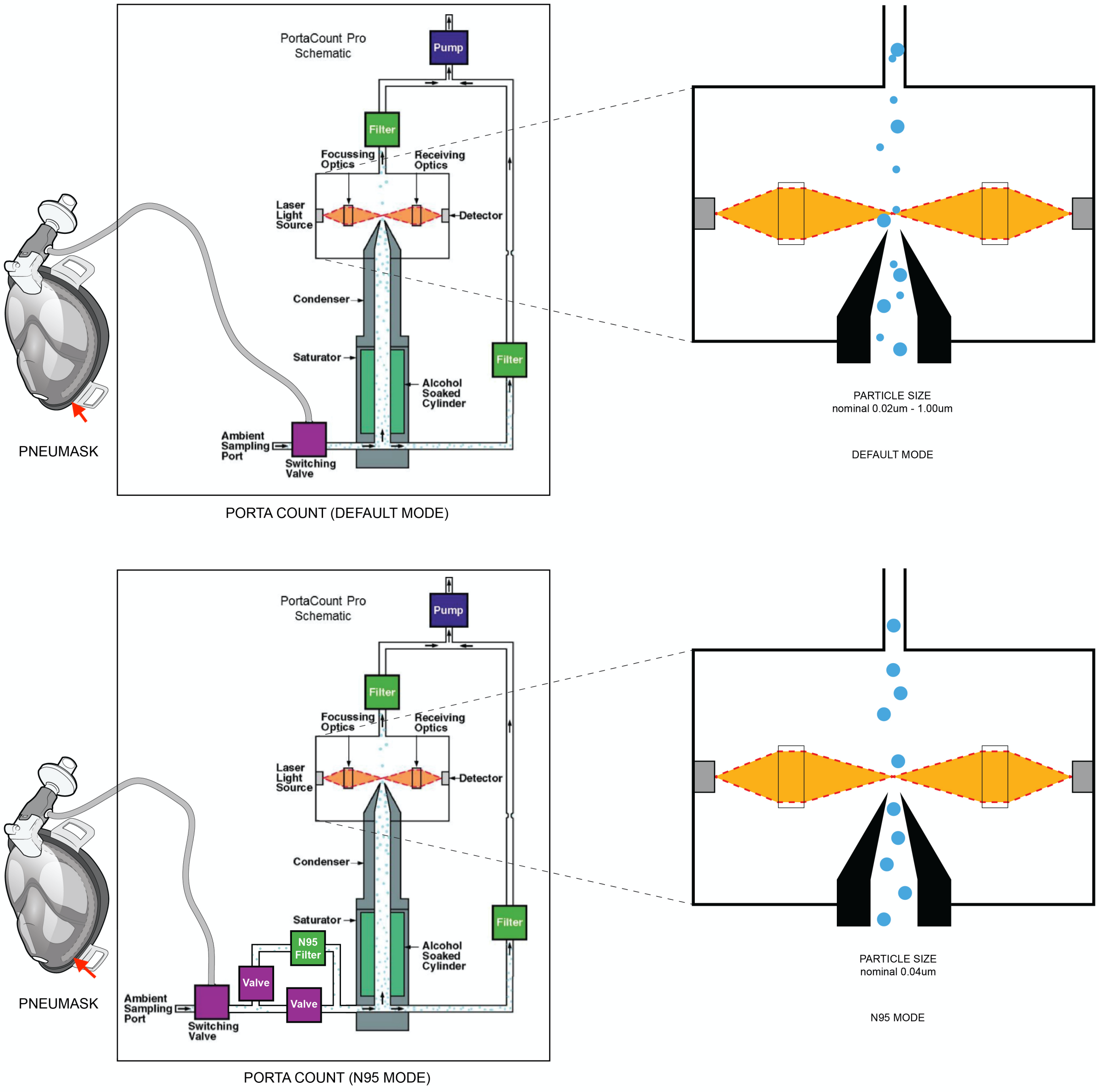

Figure 6 Difference in PortaCount's functionality across the default and N95 modes.

Summary of Fit-Test Findings The successful results for the fit test with the different individuals used in this study indicate that the Dolfino and Decathlon masks both form acceptable seals, showing also that the custom adapter and the chin valve do not generate significant leaks. The measured fit factors correspond to the requirement for elastomeric half-mask. The remaining performance of the mask depends on the efficiency of the filter, which is attached to the mask.

The position of the sampling point gives similar results between the mouth and the eyes chambers. However, a sampling point directly connected on the custom adapter shows significant higher fit factors, which seem over evaluated, probably due to the proximity of the filter. In this case, the measured particle concentration should not be relevant of the real concentration in the breathed air.

The use of the N95 protocol of the PortaCount is important for the fit evaluation when HEPA filters are not available, especially with HME virus filters. The efficiency of the filters should be measured independently of the PortaCount system to assure safe working conditions.

1.1.2 Details about Filtration Efficiency Testing

We developed an simple experimental test rig and method for testing the particle filtration efficiency of various materials. Please note this setup is not the standard testing method which typically uses the TSI Automated filter tester 8130A. The setup pictured in Fig. 8 includes a LightHouse handheld particle counter (Model 3016 IAQ), Intex QuickFil 6C Battery Pump, a rubber stopper with 2 holes covered by 2 kim wipes to mitigate the airflow, Incense: Satya Sai Baba Nag Champa 100 Gram, connectors (universal cuff adaptor, teleflex multi-adaptor), and filters to test (Hudson RCI Main Flow Bacterial/Viral Filter, Romsons HME Disposable Bacterial Viral Filter, Pall Ultipor 25 filter). The pump with the rubber stopper, covered by 2 kim wipes, in it, provides an airflow within a range of 5.6 - 11.32 l/min to mimic that of breathing. The incense produces particles of various sizes, including those in the range picked up by the detector (0.3 μm - 10 μm). With the pump on, we measure the number of particles produced by the incense. Then we place the filter on the setup and run the particle counter to measure the number of unfiltered particles. To calculate the filtration efficiency, we calculate the ratio of unfiltered particles to the number of particles produced by the incense, and then subtract from one. The filter efficiencies for the 3 filters tested are reported in Figure 9.

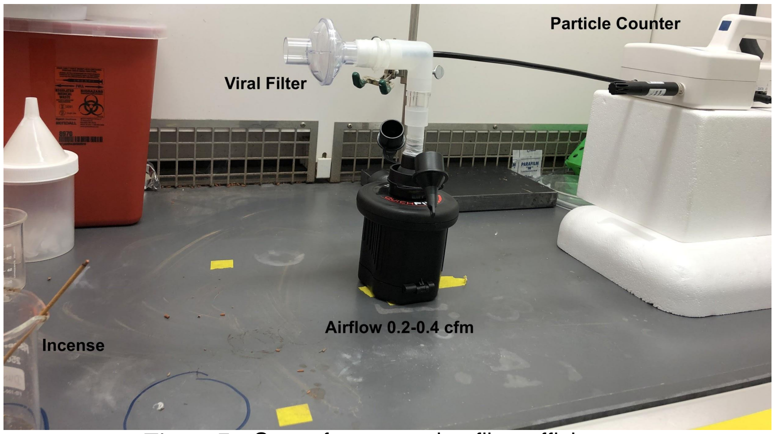

Figure 7 Setup for measuring filter efficiency.

We constructed an experimental system for measuring the pressure drop across various materials, including N95 masks, during inhalation and exhalation. The setup in Figure 10 includes an Intex QuickFil 6C Battery Pump, a Honeywell AWM700 Airflow sensor, a Honeywell ABPDLNN100MG2A3 Pressure Sensor,a rubber stopper with 2 holes covered by 2 kim wipes to mitigate the airflow, connectors (universal cuff adaptor, teleflex multi-adaptor), and filters to test (Hudson RCI Main Flow Bacterial/Viral Filter, Romsons HME Disposable Bacterial Viral Filter, Pall Ultipor 25 filter). The pump with the rubber stopper, covered by 2 kim wipes, provides an inhalation or exhalation airflow within a range of 0.2-0.4 cfm to mimic that of breathing. With the pump on, we measure the airflow applied to the mask, and the differential pressure drop across the mask. The pressure drops for the 3 filters tested are reported in Figure 9.

Summary of Filtration Testing Findings We have found that the Decathlon Subea masks and the Dolfino masks are both capable of forming a seal that exceeds the standards required for half-face respirators and N95 masks (fit factor >100). The masks must still be properly secured and sized appropriately for the wearer with the fit verified according to the standards of the institution where the PPE is being used. The sealing capabilities of these masks were tested using a TSI PortaCount Pro+ (in half-face mode using the OSHA standard) on a system that consisted of the mask, a custom adapter to connect the mask and filter, and a HEPA rated HME filter. The custom adapter was modified to include a sampling port to which the TSI PortaCount

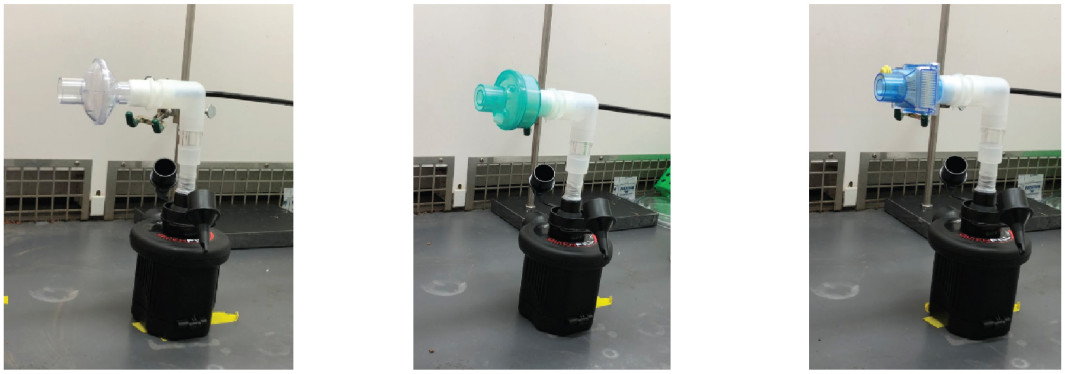

HUDSON RCI MAIN FLOW BACTERIAL/VIRAL FILTER

ROMSON HME DISPOSABLE BACTERIAL VIRAL FILTER

PALL ULTIPOR 25 FILTER

Figure 8 Variety of filters tested

Filter	Filter Efficiency of particles sized 0.3-4.49 µm for airflow rate of 2.83 l/min	Filter Efficiency of particles sized 0.3-4.49 µm for airflow rate of ~8.5 l/min	Pressure drop across filter for airflow rate of ~6.0 l/min
Hudson RCI Main Flow Bacterial/ Viral Filter	99.67% ± 0.26%	97.71% ± 1.07%	16.11 ± 1.95 Pa
Romsons HME Disposabl e Bacterial Viral Filter	99.83% ± 0.04%	97.97% ± 0.43%	18.31 ± 2.37 Pa
Pall Ultipor 25 filter	99.88% ± 0.04%	99.98% ± 0.00%	24.20 ± 4.23 Pa

Figure 9 Filter efficiency and pressure drop across filters

Pro+ could be connected, allowing measurements at three different places inside of the mask (next to the filter, in the eye space, and in the mouth space). The particle concentrations in all parts of both masks were found to be less than 1 part in 100 relative to the ambient particle concentration (Fit factor of >100). The Decathlon Subea mask was also tested at EPFL, again with a TSI PortaCount Pro+, on a system consisting of a Decathlon mask, a custom adapter, and an electrostatic HME filter. The test was run by connecting the PortaCount Pro+ to a port which was installed in the rubber siding of the mask next to the eye chamber. The test was run using an OSHA standard in N95 mode (as required by the PortaCount Pro+ for filters with <99% efficiency). The PortaCount Pro+ reported a fit factor of >200 (a particle count of less than 1 part in 200 relative to ambient conditions) for two different wearers. Repeating the test with the eye chamber and mouth chamber directly connected to allow bidirectional airflow between the two chamber resulted in the same fit factor. The sealing capability of both the Dolfino and Decathlon masks has been shown to exceed the standards for half-face respirators and N95 respirators. These tests were verified at multiple locations within Stanford and at EPFL using different masks, wearers, adapters, filters, PortaCount machines, and machine operators.

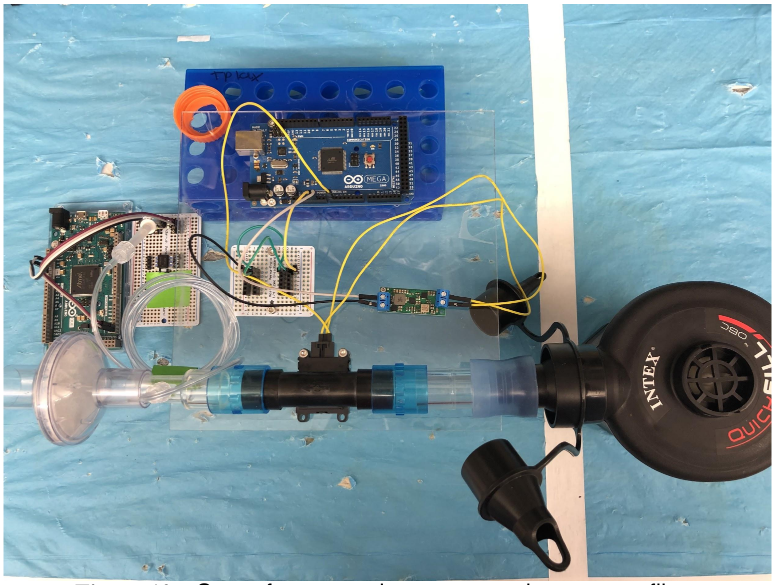

Figure 10 Setup for measuring pressure drop across filter.

1.1.3 Theoretical Estimation of Chin Valve Closure Time

Aside from the above testing, we have also done the following calculation to see how long the chin valve takes to close (Figure 11A, Figure 12), assuming standard exhale - to assess the likelihood of localized backflow. The following figure (Figure 11B) shows the schematic of a circular chin valve, which is pinned at the center. The valve is assumed to open from the bottom side, moving from vertical position to an angled location after exhalation of air.

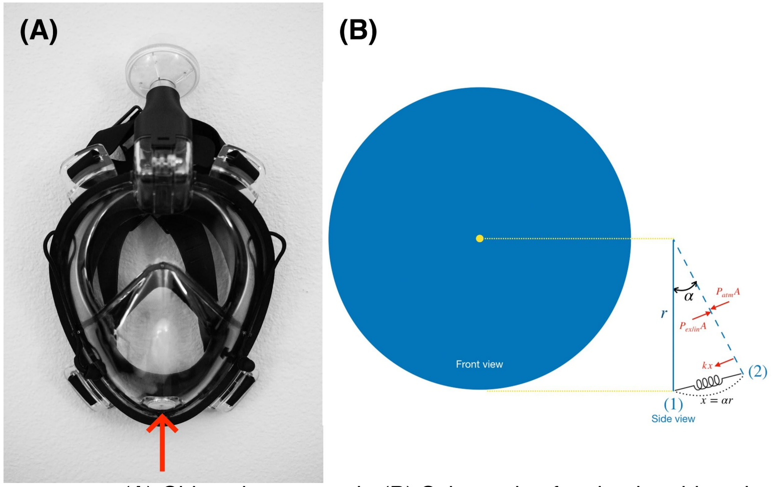

Figure 11 (A) Chin valve on mask, (B) Schematic of a circular chin valve

Forces acting on the valve include the force due to the gauge exhale pressure, ($P_{ex,g} = Pex - P_{atm}$), and elastic forces. The elastic force occurs to return the valve to its original shape, this force is simply modeled by a linear spring formula. These forces should balance for a static valve at (2):

$$P_{ex,g}A = kr\alpha \quad (2)$$

where A and k denote the cross-section area and the elasticity constant of silicon, respectively. Now, let's assume inhale starts at time t=0 and the valve is in the angled position (2) shown in Figure 11B, $\alpha = \frac{P_{ex,g}A}{kr}$

At each time instant, in addition to the elastic force, the pressure forces due to the inhale, P_{in}, and atmospheric pressure are acting on the valve:

$$\sum F = -(P_{atm} - P_{in})A - kr\theta \quad (3)$$

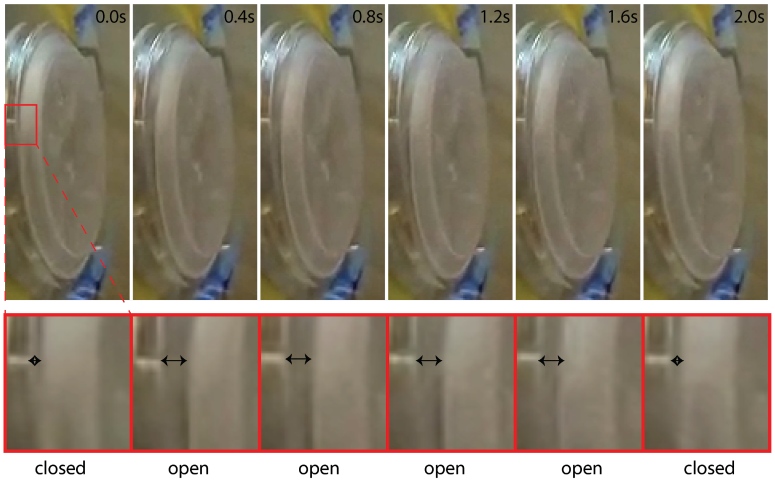

Figure 12 Time-lapse images of the chin valve opening and closing

On the other hand:

$$\sum F = ma_\theta = mr(\frac{d^2\theta}{dt^2}) \tag{4}$$

And therefore:

$$(\frac{d^2\theta}{dt^2}) + \frac{k}{m}\theta = -\frac{P_{atm} - P_{in}}{mr}A \tag{5}$$

This equation is a second-order ODE with boundary-conditions: $\theta = \alpha$ and $\frac{d\theta}{dt} = 0$ at $t = 0$.
This leads to the solution:

$$\theta = -A\frac{P_{atm} - P_{in}}{kr} + A\frac{P_{ex} - P_{in}}{kr}\cos(\sqrt{\frac{k}{m}}t) \tag{6}$$

Accordingly, the time it takes for the valve to reach $\theta = 0$ equals:

$$t = \sqrt{\frac{m}{k}}\text{acos}\frac{P_{atm} - P_{in}}{P_{ex} - P_{in}} \tag{7}$$

The maximum closure time occurs when we assume there is no force due to pressure during inhalation, $P_{in} = P_{atm}$, and equals:

$$t_{max} = \frac{\pi}{2}\sqrt{\frac{m}{k}} \tag{8}$$

In order to determine a time associated with this dynamics, we need to directly measure some of the properties of materials used in elastomeric valves. For an order of magnitude calculation, we use known numbers; for a silicon valve with density $\rho = 2.3290\text{g / cm}^3$, thickness $l = 0.35\text{mm}$, and radius 13.5mm, the mass of the moving section is $m = 0.233g$.Assuming elasticity constant $k = 1\text{N/m}$, this formula suggests that the valve closes after approximately $t_{max} = 0.024sec$.

1.1.4 Exercise Test

Currently, 3 exercise tests have been conducted by volunteers using the Pneumask-G configuration. In the first 2 tests, the volunteer was a 38 years old male, ASA 1 status, with weight of 83kg for a height of 1.80 m (2.03 m2 total body surface area by Mosteller formula). Both tests were performed at FiO2 of 21% (ambient inspired oxygen fraction at a barometric pressure of 1011 hPa).

In the first test, a Decathlon FreeBreath mask was used with a 1.6 cmH2O pressure drop, bacterial/viral filter with no HME (Heat and Moisture exchanger). The mask has been worn on a treadmill at maximum inclination, for 10 minutes, to measure the CO2 level during intense activity. For a constant running speed of 6mph, the inhaled CO2 remained below 2mmHg at all times, while the exhaled CO2 rises up to 48mmHg on peak physical effort.

The second test was performed by the same volunteer subject, in the same treadmill machine, under same general conditions for 1 hour, with the Pneumask-G configuration but with a HMEF filter with a pressure drop of 4.5 cmH2O, which is almost 3 times higher than the filter used for the first test. For the second test, we monitored heart rate, SpO2, non-invasive blood pressure (NIBP), ECG, End-tidal CO2, Inspiratory CO2, FiO2, as well as a number of subjective measures including discomfort and stamina. The results of this test are summarized in Table S??. These results indicate that the change in Inspiratory CO2 throughout use of the device, in exertion that simulates that of most healthcare work, is negligible and in line with NIOSH standards [19]. Subjective comfort/discomfort was rated from 1 of complete discomfort to 10 of complete comfort. It is notable that this never fell below a rating of a 7. Further, the volunteer had an appropriate HR response for the level of exertion and no further alterations in physiological processes were noted. This indicates the device performs similar to elastomeric respirators under near identical conditions.

1.2 Donning and Doffing Procedures

We have developed suggested donning and doffing procedures based on the recommendation of UCSF [26] and from Stanford and UCSF feedback on our prototypes. A set of suggested procedures is below (Further review of this protocol by infection disease specialists or EHS officers in hospitals is required).

1.2.1 Donning Procedures

1. Eat, drink and go to the restroom. Check everything that you need to bring into the patient's room is available.
2. Tie up your hair and remove unnecessary accessories on wrists, neck and ears.
3. Perform hand hygiene.
4. Put on a surgical cap, tucking loose hair away from the face and neck.
5. Put on the first layer of gloves.
6. Put on your gown by placing your head through the top of the opening and insert your arms into the sleeves. Tie the gown properly.
7. Carefully inspect the disinfected snorkel mask, adapter and viral filter of Pneumask.
8. (Optional if they are connected already) Serially connect the adapter and viral filter to the snorkel mask.
9. (Optional for Bluetooth user) Turn on the Bluetooth microphone. Check the connection with the smartphone. Put the Bluetooth microphone in a clean, small ziplock bag, Tape and secure the ziplock bag inside the mouth chamber without blocking the chin valve.
10. Perform a suction test: put the mask to your face, press it slightly while inhaling to create a suction between the mask and your face. The mask should suction to your face. Try smiling and see if the movement of facial muscle breaks the seal. IF MASK DOES NOT PASS SUCTION TEST, THE MASK SHOULD NOT BE USED.
11. Put on the mask fully. Adjust and tighten the straps as needed.
12. Inhaling or exhaling while closing off the top of the filter with your hand can be done for another seal check. With a good facial seal, the mask should suck down on your face with a good seal with inspiration, and not lift off face with exhalation.
13. Perform hand hygiene.
14. Place your gloved thumbs through the loops of the gown. Put on the second layer of gloves.

1.2.2 Doffing Procedures

1. In the anteroom (or the doorway if there is no anteroom), grasp the gown and pull away from your body. Carefully fold the gown into ball-shape and only touch the outer surface of the gown with gloved hands. Remove the gown and the outer gloves together. Dispose the gown and the gloves into a dedicated trash can.
2. Perform hand hygiene (you should still be wearing your inner layer of gloves).
3. Leave the anteroom.
4. Perform hand hygiene.

5. Inspect the integrity of the inner glove. If the inner glove is intact without gross contamination, proceed through the next step. If the inner glove is torn, broken, or grossly contaminated, remove the inner glove. Perform hand hygiene, and put on a new pair of gloves.
6. Lean head forward into sniff position. Loosen the straps and remove the mask without touching your face.
7. Use EPA-approved alcohol-based wipe, bleach wipe or hydrogen peroxide wipe to wipe the outer surface of the mask, adapter and filter. Dispose the wipe. The required contact time for different EPA-approved wipes can be found in Appendix A Table S5.
8. Use EPA-approved alcohol-based wipe, bleach wipe or hydrogen peroxide wipe to wipe the inner surface edges of the mask. Dispose the wipe.
9. Gently disconnect the filter from the mask. Dispose the filter if it is designed for one-time usage. If you plan to reuse the filter, follow the instructions from the manufacturer of the filter to store it properly, and DO NOT WET the surface of the filter.
10. Put the mask into a clean dedicated box.
11. Perform hand hygiene.
12. Remove the surgical cap.
13. Perform hand hygiene.
14. Remove the inner gloves.
15. Perform hand hygiene.
16. (Optional for Bluetooth user) Remove the ziplock bag which contains the Bluetooth microphone. Wipe the surface of the ziplock bag with EPA-approved wipe (Appendix A Table 5) and open the ziplock bag. Perform hand hygiene. Dump the microphone from the ziplock bag with one hand (dirty hand) onto the other hand (clean hand). Dispose the ziplock bag with your dirty hand. Place the microphone somewhere you will not forget with your clean hand. Perform hand hygiene.
17. By the end of the shift, bring the box and the mask to somewhere you can fully decontaminate it following our decontamination protocol, or hand it to hospital technicians per hospital policy. The recommendation of using disinfection wipe between patients and fully washing it after one shift is following the recommendation of NIOSH on elastomeric respirator.

1.3 Communication App User Instructions

1.3.1 Current Android app download instructions

1. Download the beta version of the app by visiting `http://kylecombes.com/app-debug.apk` on your phone.
2. Open the download. Your phone should prompt you to allow installations from unknown (i.e. non-Google Play) sources. If it does, simply follow the on-screen instructions. If no such prompt appears and the installation merely fails, do the following:
    - Allow app installations from unknown sources by going to your phone's Settings -> Security -> Unknown Sources and enable installations. This setting might have a different name, depending on your version of Android and device manufacturer.
    - Try to install the app again by re-opening the download or revisiting the link above.

1.3.2 App usage instructions

1. Connect your phone to a Bluetooth device with a microphone. We recommend using cheap Bluetooth headphones, which can be put inside a sterilized plastic bag, and placed inside the face compartment of the snorkel mask.
2. (Optional, for extra amplification) Connect an external speaker to the phone's wired headphone jack using an aux cord. If you do not do this, the phone will use its internal speakers.
3. Open the app.

4. If the app detects a Bluetooth device capable of streaming audio, it will say “Bluetooth ready.” If it does not, follow the on-screen instructions to ensure your device is connected properly.
5. Click “Start” to begin relaying audio from the microphone to the speaker.
6. You may now turn off the phone’s screen or switch to another app if you would like. If you need to switch speakers at any point, click “Stop,” do any plugging/unplugging, and then click “Start.”
7. Click “Stop” end relaying audio.

1.3.3 Troubleshooting

If the sound is playing out of the Bluetooth speaker instead of through the wired speaker, simply unplug and replug in the wired speaker.

Currently, the iOS version of this app is under development, and the Android app is pending publication in the Google Play Store. Please reach out to the provided contacts if you have any feedback, questions, or concerns.

Contacts: Emma Pan (epan549 at gmail.com), Kyle Combes (kcombes at olin.edu)

1.4 Decontamination Protocol Testing

Due to the cost and design of the full-face snorkel mask, sustainable use requires that the mask itself be reused. Thus, it will need to survive common decontamination procedures such as autoclaving or immersion in a bath of bleach or ethanol. We have performed preliminary tests in which we subjected the Dolfino Frontier mask to the conditions involved in common decontamination procedures; the mask is stated by the manufacturer to consist of either silicone or thermoplastic rubber and polycarbonate lenses.

We have developed and tested our decontamination protocols based on recommendations from the CDC [21], OSHA decontamination protocols for respirators [7], and the consensus of National Academy of Science on reusable elastomeric respirators (p. 76) [8]. From these guidelines, a simple approach could potentially be the combined usage of detergent and bleach to achieve decontamination of the snorkel masks. Besides sodium hypochlorite, there are other hospital-used disinfectants that meet the EPA’s criteria for use against SARS-CoV-2 [9] or CDC guidelines on chemical disinfectant use [10]. Among these, some hydrogen peroxide solutions, such as Accelerated Hydrogen Peroxide, offer the advantage of potentially being less harmful to the user and equipment, while only requiring a short contact time of just a few minutes. Ethylene Oxide sterilization is another commonly used method to disinfect heat sensitive equipment [11]; however, it requires specialized equipment and facilities, and whether access to such services, with the required turnaround time, is widely available to health institutions needs to be determined.

1.4.1 Autoclaving

We first performed a preliminary test to check whether mask functionality survives over the course of multiple autoclaving cycles. Before autoclaving the mask, we first took reference photos of its condition (Figure S2). We then autoclaved the mask using a 30 minute gravity cycle at 121 deg C and 15 psi, with 10 minutes of warm-up before sterilization and 30 minutes of drying afterwards. Afterwards there was a mild “hot plastic smell”. Small scratches were found upon visual inspection of the black plastic material. After letting the mask rest for at least 30 minutes to cool down, we again autoclaved the mask for another identical 30 minute gravity cycle. The mask survived both cycles of autoclaving without damage. Finally, we again let the mask rest for at least 30 minutes to cool down and then autoclaved the mask for another identical 30 minute gravity cycle. After this third round of autoclaving, with a cumulative autoclaving time of 90 minutes, the silicone rubber of the mask strap and mask seal appeared to remain elastic and functional. Mask was worn after autoclaving with no apparent loss of function.

1.4.2 Bleach Immersion

Besides autoclaving, the mask may be immersed in a bath of bleach for decontamination [12]. Thus, we tested whether a mask could survive the relatively harsh chemical conditions of immersion in a bath of bleach. We immersed a new snorkel mask for 10 hours in a bath of 10% bleach. There was no apparent damage afterwards despite some white coating which can be easily washed off (Figure S3). We thus concluded that our mask should be able to survive most bleach disinfection protocols used in the hospital [7].

1.4.3 Ethanol Immersion

Besides autoclaving and immersion in bleach, the mask may be immersed in a bath of ethanol for decontamination [12]. Thus, we tested whether a new mask could survive immersion in a bath of 70% ethanol for 10 hours (Figure S4). No apparent damage was noted afterwards. (Note that we should always use 95% ethanol to make the 70% ethanol solution, since 100% ethanol may contain trace amounts of benzene which is carcinogenic.)

1.4.4 Stretch Test

With our three snorkel masks treated under three different decontamination conditions (3 cycles of autoclaving, 10 hours of bleach immersion, and 10 hours of ethanol immersion, respectively), we then performed a simple stretch test on the elastomer bands of each mask by holding the strap with both hands such that the thumbs touched each other at the tips, then pulling and qualitatively observing the separation. The straps for the ethanol-treated mask appeared to have stretched the most, while the straps for the bleach-treated mask appeared to have stretched the least. Nonetheless, all the masks are functional and seal well after all the cleaning processes.

1.4.5 Dry Heat at 65C

Several reports indicate that dry heat at 65 degrees C is capable of killing any viral particles [13, 14]. Although we did not explicitly test this protocol, given the fact that the masks survived 121 degrees C in the autoclave for 30 minutes, we can safely infer that our mask will also survive a dry heat disinfection protocol.

1.5 Decontamination Summary - Guidelines

Based on above testing results and the recommendation from OSHA [7], we developed suggested protocols for cleaning and decontaminating our snorkel mask (Dolfino Frontier), which is available below. Please note that this protocol is not formally approved, and each hospital should consult their EH&S officers or infection disease specialists for a standard operating procedure. If you are using disinfectants other than bleach, please also check this table compiled by EPA for recommended cleaning time.

Another important note is that not all snorkel masks can tolerate the decontamination. For example, snorkel masks from Animdive, Tinmiu and Keystand cannot withstand the temperature of autoclave or industrial washers commonly used in OR. If you are using snorkel masks other than Dolfino Frontier, please perform appropriate testing before usage.

1.6 Suggested Decontamination Protocols

1. Perform hand hygiene.
2. Put on a gown, gloves, and a protective mask.
3. Wipe the surface of the filter with 70% ethanol or hydrogen peroxide, and carefully remove the filter.
4. Discard the wipe as biohazardous waste. Discard the filter if it is intended for single-use only. If you plan to reuse the filter, follow the instructions from the manufacturer of the filter to store it properly, and DO NOT WET the surface of the filter.
5. Wash mask and filter adapter thoroughly in warm water (43 deg C [110 deg F] maximum) with a mild detergent. A sponge that won't scratch may be used to facilitate the removal of dirt. Make sure to clean all the valves to avoid potential clogged valves.
6. Rinse the mask and adapter thoroughly in clean, warm (43 deg C [110 deg F] maximum), running water. Drain.
7. Prepare a 50ppm chlorine solution: add one milliliter of laundry bleach per liter of warm water (43 deg C [110 deg F] maximum). Prepare enough solution to fully immerse the entire mask. Remove all the air in the side channel to ensure full immersion. Please note that mixing bleach solutions with detergents can generate toxic substances. Make sure to wash out all the detergents before immersing the mask and adapter into bleach.
8. Immerse the mask and adapter in the hypochlorite solution (50 ppm of chlorine) for 2 minutes.
9. Wearing fresh gloves, rinse the mask and adapter thoroughly in clean, warm, running water (43 deg C [110 deg F] maximum). The mask and adapter must be thoroughly rinsed with water to remove any detergents or disinfectants that may result in dermatitis or damage of the mask components.
10. Dry the mask and adapter with a clean lint-free cloth or allow to air dry.
11. Reassemble the mask and store in a clean space.

1.7 Failure Modes and Effects Analysis (FMEA)

We have also performed a failure modes and effects analysis on our Pneumask-G design. In this analysis, we first decomposed the product into different components and listed the primary functions of each component. We then analyzed what would happen if each component fail to serve their primary functions and how much negative impact it would bring. By considering the severity, chance of occurrence, and chance of detection of that malfunctioning scenario, we can compute a semi-quantitative score. By comparing the semi-quantitative score of each possible failure mode, we can identify the most important failure modes that require immediate action or improvement in design.

The analysis suggested several points that may be useful for anyone to further build upon our system: (1) Using surgical hood or any additional coverage to protect the filter surface, lateral side of the mask and the straps from gross contamination may provide additional protection (2) Separating the airflow pathway for inhaled air and exhaled air as much as possible can further improve performance for the Pneumask-G design. Modification or blocking of the chin valve would require complete redesign of airflow pathway. (3) Use a single-usage filter if available. For filters designed for repetitive usage, follow the instructions and regulatory-approved extended use claims (EUC) of the manufacturers (4) Avoid prolonged usage if possible (5) Long-term durability of the adapter when subjected to many cleaning process cycles will likely be dependent on the exact material and manufacturing process. Please perform appropriate further failure testing to characterize usage lifetime of these adapters in the material that is used. (6) Instructions on cleaning should specifically mention the necessity of cleaning the valve, as discussed in step 5 of the decontamination protocol in the subsection above. (7) Use of voice amplifying system may help to minimize risk due to jaw movements (8) Encourage face washing after doffing.

2 Supplementary Tables

Company	Product Names	Filtration efficiencies
Teleflex	Main Flow Bacterial/Viral Filter (Item code 1605)	Bacterial 99.999%, viral 99.99%
	Aqua+®F Bacterial/Viral Filter (Item code 1577)	Bacterial 99.9%, viral 99.9%
	ISO-Gard® HEPA Light with port (28002)	Bacterial and viral 99.9999+%
	ISO-Gard® HEPA Light (28012)	Bacterial and viral 99.9999+%
	ISO-Gard® HEPA Light Machine (28022)	Bacterial and viral 99.9999+%
	ISO-Gard® HEPA Small, angled (28052)	Bacterial and viral 99.9999+%
	ISO-Gard® HEPA Small, straight (28062)	Bacterial and viral 99.9999+%
	ISO-Gard® Depth Filter (19212)	Bacterial 99.9999%, viral 99.99+%
	Gibeck® Humid-Vent® HMEF Series (11012, 18502, 19502, 18402, 19402, 18832, 19932, 19932-BPW)	Bacterial 99.9999%, viral 99.99+%
	GIBECK ISO-GARD (19211) - Resistance 16 mmH2O @ 60 l/min	Bacterial and viral 99.999%
	GIBECK ISO-GARD depth filter (19212) - Resistance 20 mmH2O @ 60 l/min	Bacterial 99.999% ; viral 99.99+%
Philips	Bacterial Filter (Item code 342077)	Not available on website
	Bacterial Filter (Item code 1014047)	Not available on website
AG Industry	AG7178 Final Bacterial/Viral Filter	Not available on website
Medtronic	Puritan Bennett™ Disposable Pediatric-Adult Expiratory Filtration System (out of stock)	Bacterial and viral 99.999%, N100 equivalent
	Puritan Bennett™ Re/X800 Expiratory Bacterial Filter (out of stock)	Bacterial and viral 99.999%, N100 equivalent
	Puritan Bennett™ Neonatal Expiratory Filtration System (out of stock)	Bacterial and viral 99.99%, N100 equivalent
	Puritan Bennett™ Reusable Pediatric-Adult Expiratory Filter (out of stock)	Bacterial and viral 99.999%, N100 equivalent
	Puritan Bennett™ Re/X700 Expiratory Filter (out of stock)	Bacterial and viral 99.97%, N100 equivalent
	Puritan Bennett™ Expiratory Filter (available)	Bacterial and viral 99.97%, N100 equivalent
	Puritan Bennett™ D/X800 Expiratory Filter (out of stock)	Bacterial and viral 99.999%, N100 equivalent
Airon	Bacterial/Viral Filter (P/N 58210)	Not available on website
PALL Corporation	Pall BB50T Breathing circuit filter (Item code BB50T)	Bacterial and viral 99.9999%
	Ultipor® 25 Filter With Monitoring Port (Item code BB25AB)	Bacterial and viral 99.999%
GE Healthcare (filters not available now as confirmed in email)	HMEF Neonatal bacterial viral HMEF + Luer Port (2106570-006)	Bacterial and viral >99.9%
	Pediatric bacterial viral HMEF + Luer port (2106570-009)	Bacterial and viral 99.99%
	Adult bacterial viral HMEF + Luer port (2106570-010)	Bacterial and viral 99.999%
	Adult HEPA pleated paper hydrophobic bacterial viral filter + Luer port (2106570-008)	Bacterial and viral 99.99999%
	Adult bacterial viral filter + Luer port (2106570-007)	Bacterial and viral 99.999%
DAR	Mechanical Filter (Small)	Bacterial and viral 99.999%
GVS Filter Technologies	Electrostatic Filter without gas sampling port	Bacterial and viral 99.999%
	Mechanical HEPA filter Long Term anesthesia-ventilation	Bacterial 99.999% - viral 99.997%
	Reusable Hepa Filter - Autoclavable up to 20 times	Bacterial and viral 99.999%
	Reusable Hepa Filter	Bacterial and viral 99.999%
	Multi Vent Barb - Multi-ventilator filter	Bacterial and viral 99.999%
	Electrostatic Filter 22M-15F-22F	Bacterial and viral 99.999%
	Bacterial-Viral filter for nebulizer therapy machine	Bacterial and viral 99.999%
	Filter for nebulizer therapy machine	Bacterial and viral 99.999%
	Maxi Pleated HEPA Filter - Resistance 142 Pa	Bacterial and viral 99.9999%
Vyaire Medical	Hygroscopic Condenser Humidifier with Filter AirLife® Vyaire Medical 5708	Bacterial: 99.997% Viral: 99.94%

Table S1: List of commercially available respiratory filters which are initially designed for ventilators.

Company	Product name	Bacterial filtering efficiency	Viral filtering efficiency	Particle size
Cummins	Nanoforce Radial Seal Filter (AF25149NF / AF27993NF)*	N/A	N/A	99.99% (with 0-5 um dust per ISO 5011)
MoreWine	0.45um Filter Sheets*	99.99%	N/A	0.45 um
Nispira	HEPA Filter Replacement For Tower Air Purifier	N/A	N/A	99.97% for 0.3um
	A True HEPA Filter for Germ Guard Air Purifier AC4100 Series*	99.7%	N/A	N/A
	A True HEPA Filter for Germ Guard Air Purifier AC5000 Series*	99.7%	N/A	N/A
	8 HEPA Filters Replace Neato Botvac Vacuum part 945-0123	N/A	N/A	0.3um, efficiency N/A
	A True HEPA Filter for Guard Air Purifier AC 4800 Series*	99.7%	N/A	N/A
Carkio	HEPA Filter Replacement Part for Bosch Gas 18V-Li 14.4v Vacuum Cleaner Accessories*	N/A	N/A	0.3um, efficiency N/A
Exit15	Impresa Products 50-Pack Mushroom Growing Bags Mushroom Spawn Bags, Extra Thick 6 Mil Bags, Large Size 6" X 5" X 20" 0.2 Micron Filter Breathable, Autoclavable Bags That Stand Up*	N/A	N/A	0.2um, efficiency N/A
Aerofeel	S230 M6 polyester filter in made-to-measure panels	N/A	N/A	0.4um, efficiency 60-80%
Dyson	Dyson Tower Purifier Replacement Filter - 968126-03*	N/A	N/A	0.um, efficiency 99.97%
GVS Filter Technologies	GVS BIOP3 Filter	EN143 + certification for infectious agents; certified for the protection against biological agents and P3 - PPE in III category.		
Beach	Disposable Hygroscopic Breathers	Varies based on model selected. See link for chart on filtration efficiencies by model.		

Table S2: List of commercially available filters which are not initially designed for medical usage. More characterizations of the filtering abilities are required before using in a hospital setting. The star marks next to the product name indicate the ones Prakash Lab is planning to test. (N/A = not available.)

**Brand**	**Model** (with link)	**Size range**	**Price (retail)**
Cressi	Duke Dry	S/M, M/L	79.95
Easy Snorkel	PRODIGY	S/M, L/XL	35.99
Greatever	G2	S, L	36.99
Guardian	Ocean See	S/M	69.95
Head	Sea Vision	S/M, L/XL	64
Head by Mares	Seaview	XS/S, S/M, L/XL	54.99
HKJB	Full Face K2	XS, S, L	39.98
Jogoo	(180° View Snorkel Mask)	S/M, L/XL	19.99
Meduzi	Seafin	?	?
Midry	Midry	?	39.97
Ocean Reef	Aria Classic	S/M, M/L	69.95
Ocean Reef	Aria Junior	n/a	49.95
Ocean Reef	Aria QR+	S/M, M/L, L/XL	89.95
Ocean Reef	Uno	S/M, L/XL	60
OctoberMoon	(180° snorkel mask)	?	?
Outdoor Master (dba Greatover)	(180° snorkel mask)	?	?
QingSong	(Full face snorkel mask)	S/M, L/XL	35.99
Seabeast	AF90	S/M, L/XL	59.99
Seac USA	Magica	Junior, XS/S, S/M, L/XL	46
Seac USA	Unica	Junior, XS/S, S/M, L/XL	69
Seac USA	Libera	Junior, XS/S, S/M, L/XL	79
Swordfish Sport	Fiji	S/M, L/XL	76
Tribord (brand owned by Decathlon S.A.)	Easybreath	XS, S/M, M/L	89.9
Trimagic	(180° Full face Snorkel mask)	XS, S, M, L, XL	24.99
U.S. Divers	Airgo LX	S, M, L	69.99
Vaincre	(180° Full Face Snorkel Mask)	S/M, L/XL	24.99
Wet Products	(Adult Full Face Snorkel Mask)	n/a	59
WildHorn Outfitters	Seaview 180° V2	XS, S/M & M/L	79.99
WildHorn Outfitters	Seaview 180°	S/M, L/XL	
X-Lounger	(Snorkel Mask Full Face Foldable)	M, L	27.96

Table S3: List of commercially available full-face snorkel masks

Company	Product name	Special Notes
3M	3M™ Particulate Filter 2091/07000(AAD), P100 100 EA/Case	User manual
	3M™ Particulate Filter 2096, P100, with Nuisance Level Acid Gas Relief 100 EA/Case	
	3M™ Particulate Filter 2097/07184(AAD), P100, with Nuisance Level Organic Vapor Relief 100 EA/Case	User manual
	3M™ Advanced Particulate Filter 2291, P100 100 EA/Case	
	3M™ Advanced Particulate Filter 2296, P100, with Nuisance Level Acid Gas Relief 100 EA/Case	
	3M™ Advanced Particulate Filter 2297, P100, with Nuisance Level Organic Vapor Relief, 100 EA/Case	
	3M™ Particulate Filter 7093, P100 60 EA/Case	
	3M™ Particulate Filter 7093B, P100 144 EA/Case	
	3M™ Hydrogen Fluoride Cartridge/Filter 7093C, P100, with Nuisance Level Organic Vapor and Acid Gas Relief, 60 EA/Case	
	3M™ Organic Vapor Cartridge/Filter 60921, P100 60 EA/Case	
	3M™ Organic Vapor/P100 Service Life Indicator Cartridge 60921i	
	3M™ Acid Gas Cartridge/Filter 60922, P100 60 EA/Case	
	3M™ Organic Vapor/Acid Gas Cartridge/Filter 60923, P100, 60 EA/Case	
	3M™ Ammonia/ Methylamine/Filter 60924, P100, 60 EA/Case	
	3M™ Formaldehyde Organic Vapor Cartridge/Filter 60925, P100 60 EA/Case	
	3M™ Multi Gas/Vapor Cartridge/Filter 60926, P100 60 EA/Case	
	3M™ Organic Vapor/Acid Gas Cartridge/Filter 60928, P100 60 EA/Case	
	3M™ Mercury Vapor/Chlorine Gas Cartridge/Filter 60929S, P100, 60 EA/Case	
MSA	Flexi-Filter P100 818342	
	Flexi-Filter P100 with nuisance level AG, HF Removal 818344	
	Flexi-Filter N95 818346	
	Flexi-FIlter N95 with Nuisance Level OV Removal 818347	
	Advantage Chemical and Combination Cartridges P100 10123079	
	Cartridges, ADV, GMA-P100 815362	
	Cartridges, ADV, GMB-P100 815363	
	Cartridges, ADV, GMC-P100 815364	
	Cartridges, ADV, GMD-P100 815365	
	Advantage Cartridges, Multigas/P100(GME) 815366	
	Cartridges, ADV, MERSORB-P100 815368	
	Cartridges, Advantage, GMI-P100 815641	
	Comfo Respirator Cartridge, Low-Profile P100 10153412	
	Cartridges, Comfo, Sprkfoe, P100 815176	
	Cartridges, Comfo L-profile, P100 815177	
	Cartridges, Comfo, GMA-P100 815178	
	Cartridges, Comfo, GMB-P100 815179	
	Cartridges, Comfo, GMC-P100 815180	
	Cartridges, Comfo, GMD-P100, 815181	
	Cartridges, Comfo, GME-P100 815182	
	Cartridges, GMI-P100 815184	
	Cartridges, Comfo, Mersorb-P100 815185	
	Cartridges, Comfo, GMA-P100 815186	
	Cartridges, Comfo, GMC-P100 815188	
Honeywell	Honeywell P100 Filter Replacement Kit, For Honeywell Convenience Pack Respirators, RWS-54042	Product guide

Table S4: List of commercially available P100 respirator filters/cartridges originally for industrial usage.

Active Ingredient	Product Name	Company	Testing Viruses	Contact Time(min)	Emerging pathogen claim
Sodium hypochlorite	Micro-kill Bleach Germicidal Bleach Wipes	Medline Industries Inc	Norovirus	0.5	Yes
Hydrogen peroxide	Oxy-1 Wipes	Virox Technologies Inc	Poliovirus	0.5	Yes
Quaternary ammonium; Isopropanol	Discide Ultra Disinfecting Towelettes	Palermo Healthcare LLC	Human coronavirus	0.5	No
Hydrogen peroxide; Peroxyacetic acid	Peridoxrtu (Brand) One-step Germicidal Wipes	Contec Inc	Human coronavirus	0.5	No
Quaternary ammonium; Ethanol	Opti-cide Max Wipes	Micro-Scientific LLC	Rotavirus	1	Yes
Sodium hypochlorite	Sani-Cloth Bleach Germicidal Disposable Wipe	Professional Disposables International Inc	Adenovirus; Rotavirus; Canine parvovirus; Hepatitis A virus; Poliovirus Type 1; Rhinovirus Type 37; Feline calicivirus	1	Yes
Hydrogen peroxide	Accel TB Wipes	Virox Technologies Inc	Poliovirus	1	Yes
Hydrogen peroxide	Oxivir™ Wipes	Diversey Inc	Norovirus; Poliovirus Type 1; Rhinovirus Type 14	1	Yes
Hydrogen peroxide	Oxivir 1 Wipes	Diversey Inc	Enterovirus Type D68	1	Yes
Quaternary ammonium; Ethanol	Peak Disinfectant Wipes	North American Infection Control Ltd	Poliovirus Type 1; Rhinovirus	1	Yes
Sodium hypochlorite	Avert Sporicidal Disinfectant Cleaner Wipes	Diversey Inc	Human coronavirus	1	No
Quaternary ammonium; Isopropanol	Super Sani-Cloth Germicidal Disposable Wipe	Professional Disposables International Inc	Rhinovirus 39; Adenovirus	2	Yes
Hydrogen peroxide	Clorox Commercial Solutions® Hydrogen Peroxide Cleaner Disinfectant Wipes	Clorox Professional Products Company	Norovirus	2	Yes
Quaternary ammonium	Nugen 2m Disinfectant Wipes	Lonza LLC	Human coronavirus	2	No
Quaternary ammonium	Caterpillar	Reckitt Benckiser	Human coronavirus	2.5	No
Quaternary ammonium; Isopropanol	Opti-Cide 3® Wipes	Micro-Scientific LLC	Rotavirus; Rhinovirus	3	Yes
Quaternary ammonium	AF3 Germicidal Disposable Wipe	Professional Disposables International Inc	Rotavirus; Adenovirus	3	Yes
Quaternary ammonium; Ethanol; Isopropanol	Caviwipes 1	Metrex Research	Adenovirus	3	Yes
Sodium hypochlorite	Caviwipes Bleach	Metrex Research	Feline calicivirus	3	Yes
Quaternary ammonium	PJW-622	Clorox Professional Products Company	Rotavirus	3	Yes
Sodium hypochlorite	Clorox Healthcare® Bleach Germicidal Wipes	Clorox Professional Products Company	Canine Parvovirus; Feline Parvovirus	3	Yes
Quaternary ammonium; Ethanol; Isopropanol	Sani-Cloth Prime Germicidal Disposable Wipe	Professional Disposables International Inc	Feline Calicivirus	3	Yes
Quaternary ammonium	Sani-cloth Germicidal Disposable Cloth	Professional Disposables International Inc	Human coronavirus	3	No
Quaternary ammonium	Clorox Disinfecting Wipes	The Clorox Company	Rotavirus	4	Yes
Quaternary ammonium	Clorox Commercial Solutions® Clorox® Disinfecting Wipes	Clorox Professional Products Company	Rotavirus	4	Yes
Quaternary ammonium	Lonza Disinfectant Wipes Plus	Lonza LLC	Human coronavirus	4	No
Quaternary ammonium	Nugen Low Streak Disinfectant Wipes	Lonza LLC	Human coronavirus	4	No
Quaternary ammonium	Clorox Healthcare® VersaSure® Wipes	Clorox Professional Products Company	Norovirus	5	Yes
Ethanol	PURELL Professional Surface Disinfectant Wipes	GOJO Industries Inc	Norovirus	5	Yes
Phenolic	Sporicidin (Brand) Disinfectant Towelettes	Contec Inc	Human coronavirus	5	No

Table S5: List of EPA-certified wipes. It is ordered by required contact time. All listed products are approved for healthcare use. If a product qualifies for the emerging viral pathogen claim, it is effective against a harder-to-kill virus than human coronavirus. All products on this list meet EPA's criteria for use against SARS-CoV-2, including those marked as "No" in this column.

Time (Minutes)	5	10	15	20	25	30	35	40
Heart Rate	85	76	84	81	93	85	88	72
Respiratory Rate	13	13	13	15	13	19	16	14
SatO2%	97 %	96 %	96 %	96 %	98 %	96 %	95 %	95 %
ECG	NSR	NSR	NSR	NSR	NSR	NSR	NSR	NSR
MAP	78	95	97	97	92	90	89	87
Systolic Pressure	101	127	116	125	113	119	114	114
Diastolic Pressure	67	70	67	77	73	68	70	71
EtCO2	38	40	45	41	40	41	40	42
InspCO2	6	7	9	8	8	9	7	8
FiO2	21 %	21 %	21 %	21 %	21 %	21 %	21 %	21 %
VAS scale (for Headache if any)	0	0	0	0	0	0	0	0
Dispnea (0-10)	0	0	0	0	0	0	0	0

Time (Minutes)	45	50	55	60	65	70	75	80
Heart Rate	80	70	78	68	62	63	65	85
Respiratory Rate	12	9	10	14	12	16	14	12
SatO2%	96 %	96 %	96 %	92 %	96 %	96 %	97 %	97 %
ECG	NSR	NSR	NSR	NSR	NSR	NSR	NSR	NSR
MAP	94	95	85	79	78	84	76	80
Systolic Pressure	117	121	113	108	103	109	101	109
Diastolic Pressure	72	77	70	59	62	56	54	55
EtCO2	42	41	42	40	42	40	42	41
InspCO2	8	7	3	1	8	4	3	4
FiO2	21 %	21 %	21 %	21 %	21 %	21 %	21 %	21 %
VAS scale (for Headache if any)	0	0	0	0	0	0	0	0
Dispnea (0-10)	0	0	0	0	0	0	0	0

Table S6. Data from CO_2 testing on Dolfino Frontier.

Time (Minutes)	5	10	15	20	25	30	35	40	45	50	55	60
Heart Rate (min^{-1})	109	100	120	133	128	131	122	135	135	134	133	140
SatO2 (%)	99	98	98	97	97	96	97	96	97	97	97	97
NIBP S (mmHg)	135	130	124	117	113	112	115	114	112	111	112	94
NIBP D (mmHg)	79	87	94	70	63	72	59	74	69	71	68	72
ECG	NSR	NSR	NSR	NSR	NSR	NSR	NSR	NSR	NSR	NSR	NSR	NSR
EtCO2 (mmHg)	41	38	43	47	45	45	43	43	41	40	45	42
InspCO2 (mmHg)	4	3	2	2	2	2	2	2	2	2	3	2
FiO2	21%	21%	21%	21%	21%	21%	21%	21%	21%	21%	21%	21%
*Subjective comfort/ discomfort	9	9	9	8	8	7	7	7	6	6	6	6
**Subjective Stamina	10	10	9	9	8	7	7	7	6	6	6	6
Speed KPH	3	5	5	5	6	5	5	5	5	5	5	5
Inclination (º)	5	10	10	10	10	10	10	10	10	10	10	10
Room Temperature	N/A	N/A	N/A	N/A	N/A	N/A	N/A	N/A	N/A	N/A	N/A	N/A
Room Humidity	N/A	N/A	N/A	N/A	N/A	N/A	N/A	N/A	N/A	N/A	N/A	N/A
Time (Minutes)	5	10	15	20	25	30	35	40	45	50	55	60
Fogging (0-10)	0	0	0	0	1	2	2	2	3	3	3	3

Table S7. Results of the 1 Hour treadmill test.

Filtration Method	HMEF?	Machine/Expiratory or Patient	Product Family (Company)	Part Numbers	VFE
			Curaplex Aero-Pro HEPA Light Machine (Tri-Anim)	301-431	99.9999%
		Machine/Expiratory	BB50T (Pall)	**BB50T**	99.999%
			Air-Guard (Intersurgical)	1790000, 1790000S, 1790007	99.9999%
			DAR Mechanical Filter (Medtronic)	351U5878, 351U5856, 351U5410, 351U5979	99.9999%
			HEPA filter (GE Healthcare)	2106570-008	99.9999%
			Curaplex Aero-Pro HEPA Light (Tri-Anim)	301-439	99.9999%
			SafeStar (Dräger)	MP01790, MP01785, MP01795	99.9999%
			Medline Respiratory / Anesthesia HEPA Filters (Medline)	DYNJAAHEPA	99.9999%
			Westmed HEPA Filter (Westmed)	6159	99.9999%
			SunMed Hepa Filter (SunMed)	FH603026	99.9999%
			Ultipor 100 (Pall)	BB100A, BB100AF	99.999%
			Ultipor 25 (Pall)	BB25, BB25F, BB25AB	99.999%
	No	Patient	Hydro-Guard (Intersurgical)	1744000S, 1745197, 1755012, 1745711, 1744011S, 1	99.999%
		Machine/Expiratory	Gibeck Iso-Guard HEPA Light Machine (Teleflex Medical)	28022	99.9999%
	Partial	Patient	Gibeck Iso-Guard HEPA Light/Small (Teleflex Medical)	28002, 28012, 28052, 28062	99.9999%
			Curaplex Aero-Sat HEPA (Tri-Anim)	301-417, 301-418, 301-423, 301-424, 301-437	99.9999%
			DAR Mechanical HMEF (Medtronic)	35U5876	99.9999%
			Twinstar HEPA (Dräger)	MP01801	99.9999%
Pleated Hydrophobic or Mechanical	Yes	Patient	Medline HEPA Filtered Heat and Moisture Exchangers (Me	DYNJAAHMEHEPA	99.99%
			Flo-Guard (Intersurgical)	1690000	99.99%
		Machine/Expiratory	Main Flow Bacterial/Viral Filter (Teleflex Medical)	1605	99.99%
			Gibeck Iso-Guard Depth Filter	19212	99.9999%
			Filta-Guard (Intersurgical)	1944003, 1944011	99.999%
			DAR Electrostatic Filters (Medtronic)	350U5865, 350U5879	99.999%
			Uni-filter/S (Vyair Medical)	55702250, 8570230, M1010541, M1003345	99.999%
			Curaplex BFV/VFE Depth Filter (Tri-Anim)	301-435	99.999%
			Vyaire B/V Filters (Vyaire Medical)	5096HEPA, 303HEPA	99.999%
			Interguard (Intersurgical)	1344007S, 1344000S	99.998%
			Carestar (Dräger)	MP01770, MP01755, MP01765	99.99%
			Westmed clear bacterial/viral filter (Westmed)	6216	99.99%
			Medline Respiratory / Anesthesia BV Filters (Medline)	DYNJAABV1	99.99%
			GE Healthcare bacterial/viral filter (GE Healthcare)	2106570-007	99.99%
			Clear Guard (Intersurgical)	1544000, 1544007, 1545000, 1344711S, 1544012, 15	99.99%
			SunMed BV Filter (SunMed)	FH603003, BF103, BF102	99.99%
			Thermoflo Filter (ARC Medical)	6000	99.99%
			Thermoflo Filter Special (ARC Medical)	6000S	99.99%
			Curaplex Aero-Pro (Tri-Anim)	301-411, 301-412	99.95%
			CircuitGuard filter (ARC Medical)	7056, 7061	99.9%
	No	Patient	Clear-Guard Midi (Intersurgical)	1644000, 1644007, 1644011, 1644012, 1644137	99.9%
			Filta-Therm (Intersurgical)	1941351, 1941197, 1941011, 1941001, 1942000, 190	99.999%
			TwinStar 90 (Dräger)	MP01800	99.999%
			DAR Electrostatic Filter HME	35U5996, 35U5877, 35U5805	99.999%
			Thermoflo Midi HCH with Filter (ARC Medical)	6310SE, 6310S, 6310E, 6310	99.999%
			Vyaire HMEF 750 (Vyaire Medical)	M1010538, M1004132, M1038639	99.998%
			Inter-therm (Intersurgical)	1341351S, 1341012S, 1341974S, 1341197S, 134158	99.998%
			Westmed HME Filter (Westmed)	6220, 6218, 6379, 6221	99.99%
			Gibeck Humid-vent filter (Teleflex medical)	18402, 19402, 18832, 19932, 18502, 19502	99.99%
			TwinStar (Dräger)	MP01805, MP01810	99.99%
			Clear-Therm (Intersurgical)	1541351, 1541974, 1541012, 1541197, 1541011, 154	99.99%
			CircuitGuard HCH and filter (ARC Medical)	6126, 6131	99.99%
			GE Healthcare HMEF (GE Healthcare)	2106570-010	99.99%
			Vyaire HMEF 1000 (Vyaire Medical)	M1010534, 557070100, M1038637	99.99%
			Medline High-Efficiency Filtered Heat and Moisture Exchar	DYNJAAHME9H, DYNJAAHME9, DYNJAAHME5, DY	99.99%
			SunMed HMEF (SunMed)	FH603009, FH603005, FH603008, FH603011, FH603	99.99%
			Thermoflo 1 HCH with Filter (ARC Medical)	6121, 6101, 6120, 6100	99.99%
			Vyaire HMEF 500 (Vyaire Medical)	557070500	99.98%
			Curaplex Aero-Sat (Tri-Anim)	301-413, 301-414	99.95%
Electrostatic	Yes	Patient	Vyaire HMEF HEPA (Vyaire Medical)	5708HEPA	99.95%

Table S8. List of commercially available medical inline filters compatible with Pneumask, sorted by filter efficiency.

3 Supplementary Figures

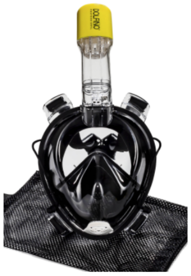	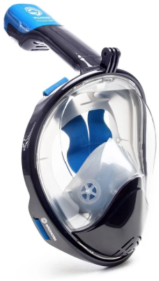	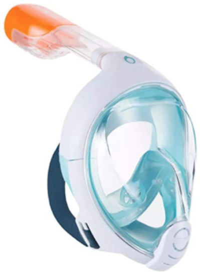	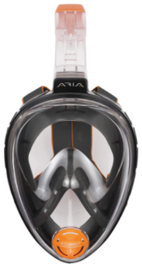	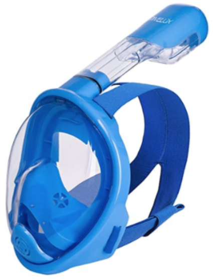
Dolfino Frontier<br>Walmart	Seaview 180 V1<br>Wildhorn Outfitters	Easybreath V1<br>Subea.fr	Ocean Reef Aria<br>Amazon US	DIVELUX<br>Amazon USNo
Tested model	To be tested	Original model	To be tested	To be tested
CAD adapter	Waiting for CAD	CAD adapter	Waiting for CAD	CAD adapter
**WMM17478SBK**				

Figure S1a Table of various full-face snorkel masks readily available in the market.

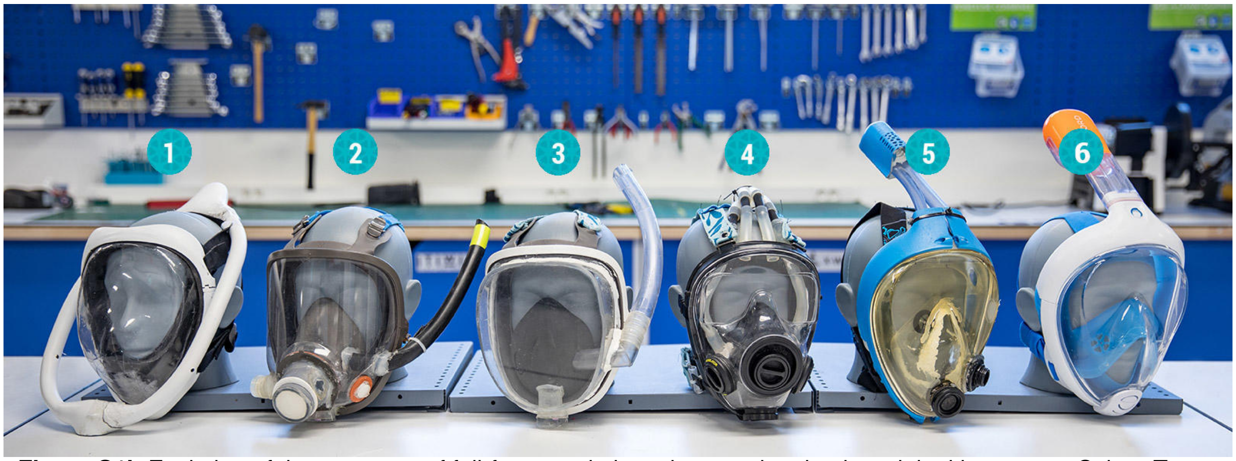

Figure S1b Evolution of the prototype of full-face snorkel masks over time by the original inventors - Subea Team.

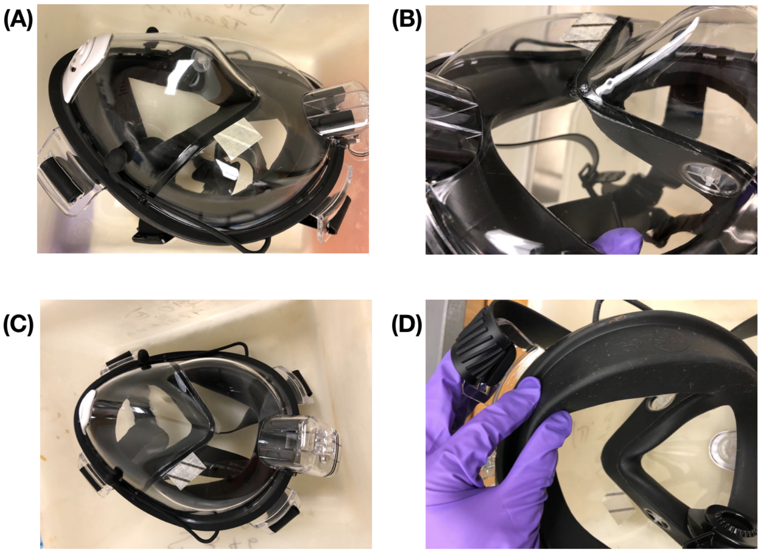

Figure S2 Before Autoclave (A), Round 1 (B), Round 2 (C) Round 3 photos and video (D). The mask survives autoclaving at 121 deg C and 15psi for a total of 90 minute cycle (done in three cycles of hot and room temperature) without apparent damage. The folders of photos and/or videos are available with the link in the caption.

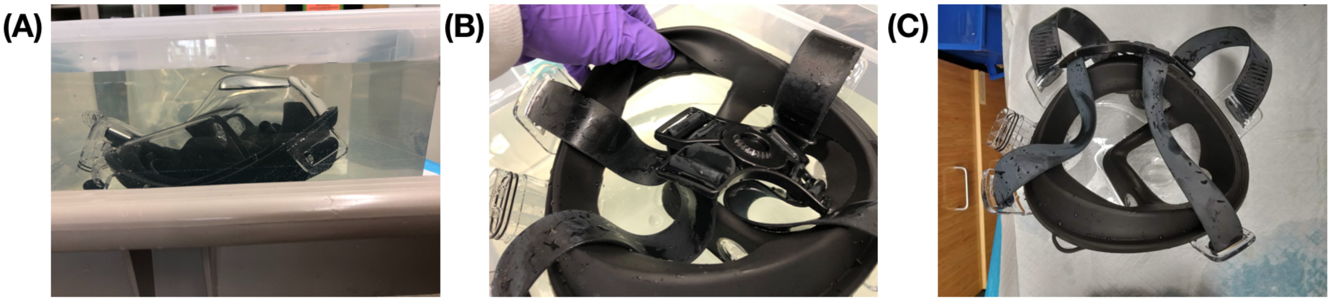

Figure S3: (A) Mask immersed in 10% bleach in a simple tupperware. (B) After 2 hours of immersion, we found no apparent damage to the mask. (C) After 8.25 additional hours of immersion, we again observed no apparent damage to the mask, though white coating had formed on the surface of the black elastomer bands.

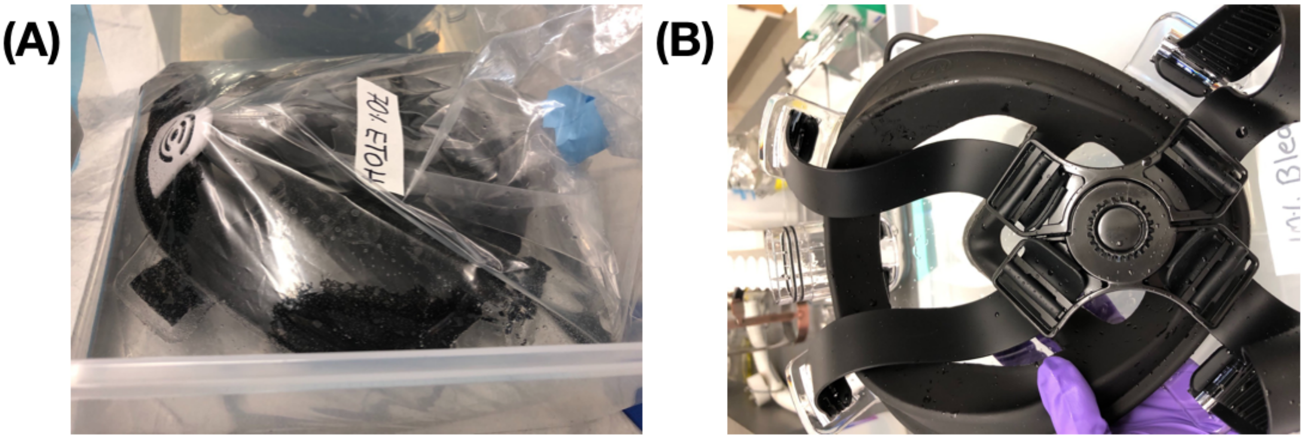

Figure S4: (A) Before (B) After 2 hours of immersion, we found no apparent functional damage to the mask.

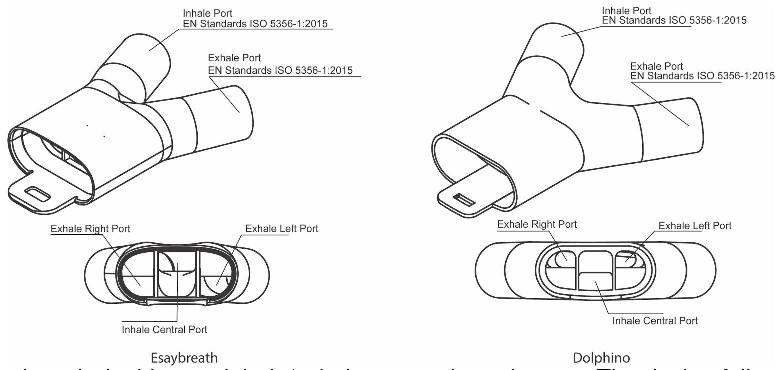

Figure S5:Dolfino and Easybreath double port inhale/exhale separation adapters. The design followed EN Standards ISO 5356-1:2015. A set of connectors ISO 5356 are available on GrabCAD thanks to Filip Kober. The CAD files of the universal adapter for Easybreath (https://a360.co/2Ukh6w5) and Dolfino Frontier (https://a360.co/39jnJCQ) are available with the links provided.

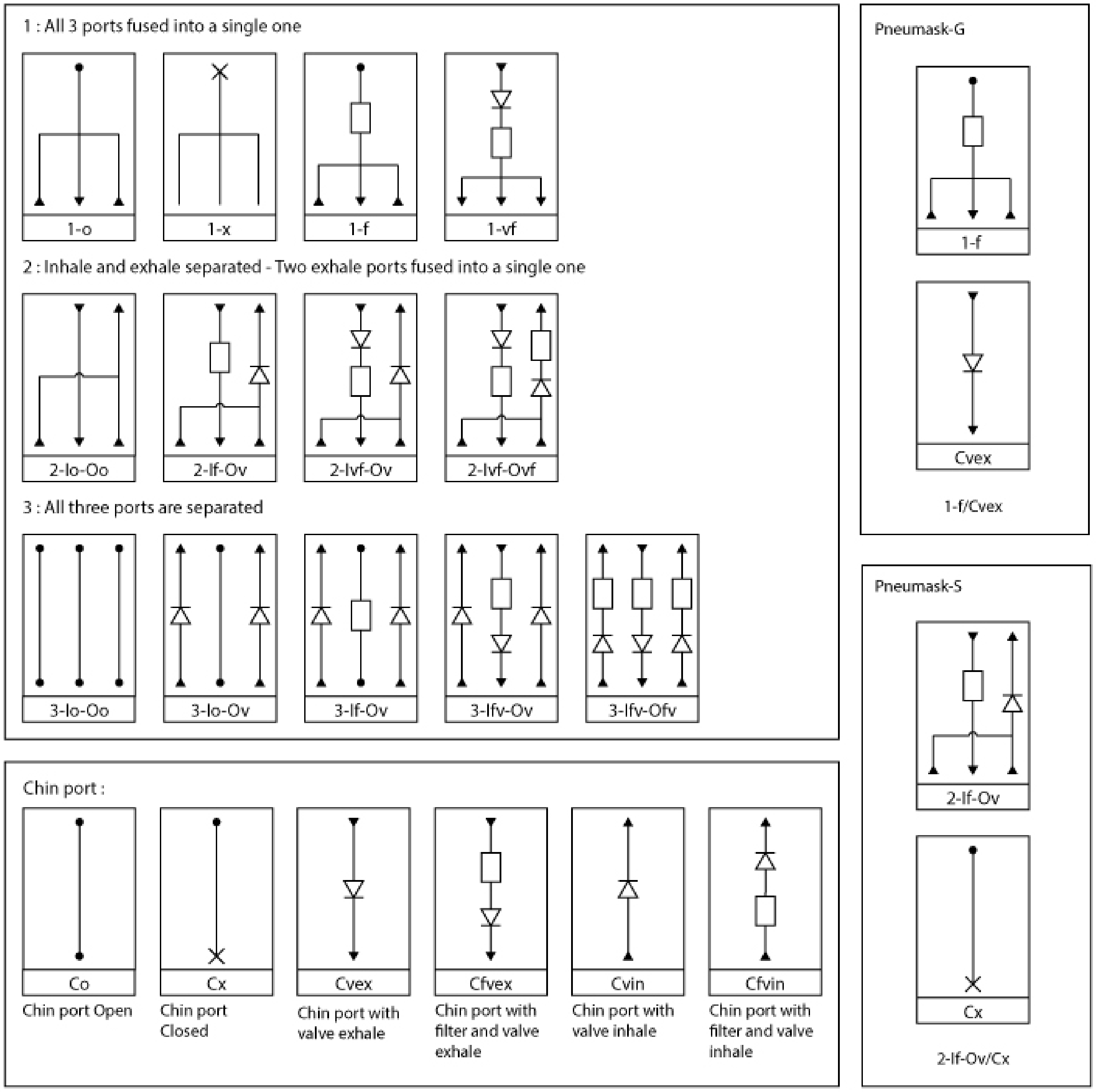

Figure S6: We have developed a nomenclature of the different combinations existing for the air flow pathway. Pneumask-G could then be named 1-f/Cvex and Pneumask-S named 2-If-Ov/Cx. Notation: diodes = one-way valves; resistor = filter. Please find the .ai original vector file here.
